# Carbon Dots as New Generation Materials for Nanothermometer: Review

**DOI:** 10.1186/s11671-020-03413-x

**Published:** 2020-09-22

**Authors:** Lazo Jazaa Mohammed, Khalid M. Omer

**Affiliations:** Department of Chemistry, College of Science, University of Sulaimani, Qliasan Street, Sulaimani City, Kurdistan Region 46002, Iraq

**Keywords:** Nanothermometer, Non-contact thermometer, Fluorescent thermometer, Carbon dots nanothermometer

## Abstract

Highly sensitive non-contact mode temperature sensing is substantial for studying fundamental chemical reactions, biological processes, and applications in medical diagnostics. Nanoscale-based thermometers are guaranteeing non-invasive probes for sensitive and precise temperature sensing with subcellular resolution. Fluorescence-based temperature sensors have shown great capacity since they operate as “non-contact” mode and offer the dual functions of cellular imaging and sensing the temperature at the molecular level. Advancements in nanomaterials and nanotechnology have led to the development of novel sensors, such as nanothermometers (novel temperature-sensing materials with a high spatial resolution at the nanoscale). Such nanothermometers have been developed using different platforms such as fluorescent proteins, organic compounds, metal nanoparticles, rare-earth-doped nanoparticles, and semiconductor quantum dots. Carbon dots (CDs) have attracted interest in many research fields because of outstanding properties such as strong fluorescence, photobleaching resistance, chemical stability, low-cost precursors, low toxicity, and biocompatibility. Recent reports showed the thermal-sensing behavior of some CDs that make them an alternative to other nanomaterials-based thermometers. This kind of luminescent-based thermometer is promising for nanocavity temperature sensing and thermal mapping to grasp a better understanding of biological processes. With CDs still in its early stages as nanoscale-based material for thermal sensing, in this review, we provide a comprehensive understanding of this novel nanothermometer, methods of functionalization to enhance thermal sensitivity and resolution, and mechanism of the thermal sensing behavior.

## Introduction

Temperature is a fundamental thermodynamic variable that has a remarkable influence on the biological and chemical systems. On account of its wide range of applications, almost in all fields of natural sciences, engineering, agricultural, and medical sciences, precise temperature determination is of great significance [1, 2]. In medical applications, thermometry is used for early detection of various diseases, such as stroke, cancer, or inflammations, one of whose incipient symptoms is the emergence of localized temperature peculiarities.

**Fig. 1:**
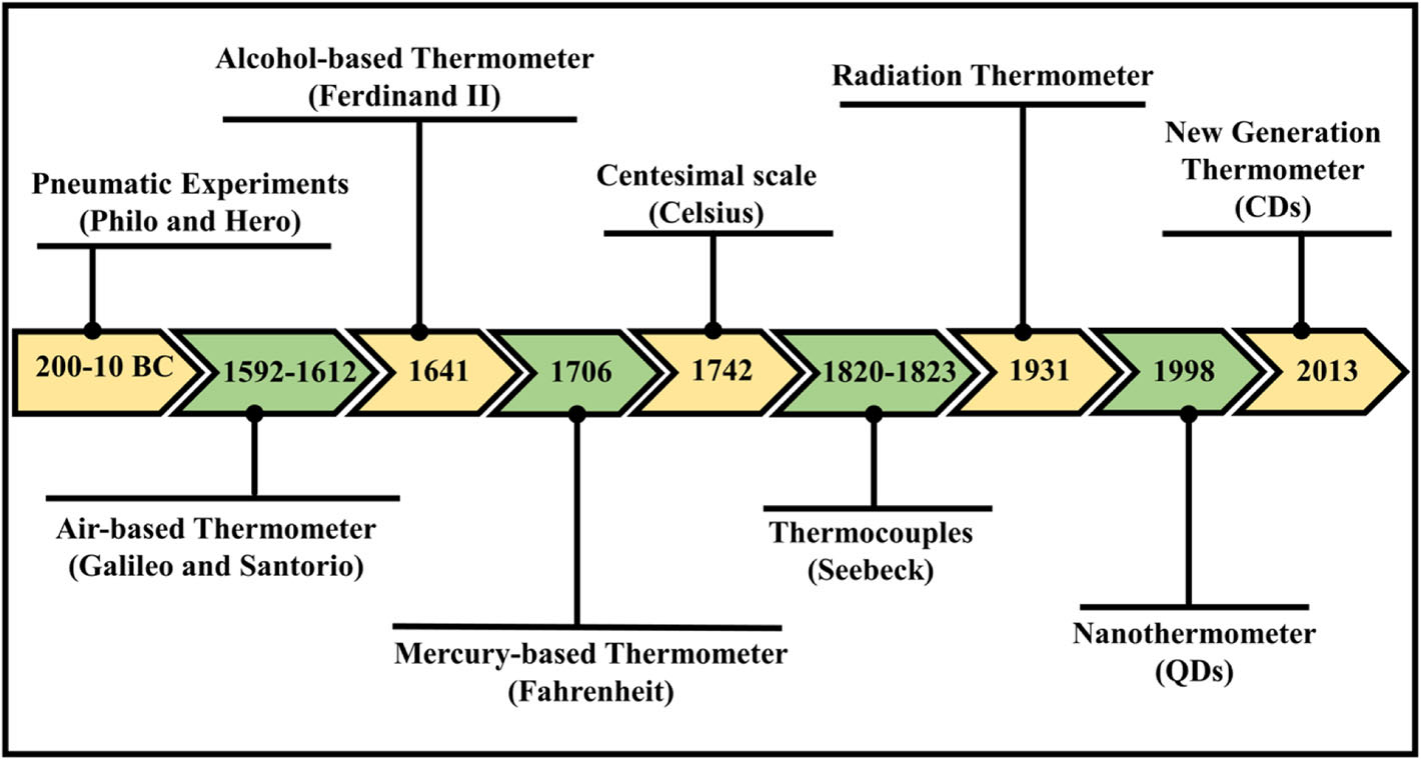
Timeline scheme for the evolution of thermometers

In history, the earliest estimation of temperature was feasibly built on sensation or observation. In ancient times, 200–10 bc, pneumatic experiments (air expansion by heat) assign as the oldest recognized references of apparatuses utilized for quantitative measurements of heat. Amid the most primitive writings related to heat expanded air are credited to the works authored by Philo of Byzantium and Hero (or Heron) of Alexandria concerning pneumatic experiments [3]. Later on, between the years 1592 to 1603, Galileo Galilei invented a thermoscope by setting experiments with the expansion of air by heat via the building of a simple apparatus using a tube encompassing air trapped above a column of water. Following Galileo, the Italian Santorio is accredited first for integrating this simple apparatus into medical examinations of fever. The first fully sealed liquid-in-glass thermometer, alcohol-filled glass tube, was assembled by Ferdinand II in the year 1641. He was able to measure temperature unaided by barometric pressure, unlike Galileo and Santorio’s open thermoscope. Fahrenheit’s practical work in thermometry emerged in 1706; he started with alcohol but subsequently became legendary for his mercury thermometers. Recognition for the centesimal temperature scale has been provided to Anders Celsius, who in 1742 projected a scale with zero at the temperature of water boiling and 100 at the temperature of water freezing. Electronic experimentations performed in the nineteenth century when Thomas Johann Seebeck considered the concept of thermoelectricity. In a succession of experiments conducted between the years 1820 and 1823, he verified the electrical potential in the juncture-points of two different metals when there is a heat difference between the joints. This was later known as the Seebeck Effect and it serves as the origin of the thermocouple which is considered the most accurate measurement of temperature [3–7]. The schematic timeline of the thermometer is shown in Fig. 1.

Conventional thermometers can be categorized into:

1. Liquid-filled glass thermometers based on the thermal expansion of materials
2. Thermocouples based on the Seebeck effect
3. Optical sensors [8]

Furthermore, they can be classified as contact or non-contact thermometers. Contact mode, including classical liquid-filled glass, thermocouples, thermistors, and resistance temperature detectors (RTDs), all necessitate electrical cabling and a direct touch between the thermometer and the substrate. This mode is not appropriate for applications in which electromagnetic noise is strong, sparks could be dangerous, the environment is destructive, or parts are promptly moving. Besides, traditional thermometers are not capable of measurements when spatial resolution lowers to the sub-micron scale, for instance, in intracellular temperature variations and mapping the temperature of microcircuits and microfluidics [9]. In the same way, engineering applications necessitate advanced thermosensitive strategies for miniatured regions and difficult environment [10]. Thus, for nano-scale domains, one should think about other approaches and materials.

Novel non-contact thermometry can overcome the aforementioned problems. For example, optical thermosensors (molecular thermometers) are a more recent generation of analytical tools that consists of molecular classes that use the measurement of emitted light to extract temperature [11, 12]. Fluorescent temperature-sensitive probes offer a promising area for thermometry in nano-system applications. The temperature information can be extracted based on their fluorescence intensity, band shape, Stokes shift, or decay lifetime can relate temperature if properly calibrated [13, 14].

Molecular thermometers have great potential in diagnosing diseased or carcinogenic cells, which have varying physiological temperatures than ordinary cells. In the medical applications, possibilities range from the temperature-induced control of gene expression [15] and cell metabolism [16] to the cell-selective penetration and treatment of disease [17] and improving the heat dissipation from integrated heat sources [18]. Recently, nanomaterials such as semiconductors [19], polymeric [10], and metallic nanoparticles [20] have been used as thermal-sensor (nanothermometer) that showed sub-micron thermal resolution.

Thermometers that can resolve sub-degree of temperature over a wide array of temperatures that also can become integrated within living systems could offer an influential new tool in countless areas of biological, physical, and chemical research. Therefore, in this review, we focus on a “new generation” or a “new class” of nanothermometer that is based on carbon nanomaterials (carbonaceous materials). To the best of our knowledge, there is no reported review article on carbon dots as a nanothermometer. Recently, carbon dots (carbon quantum dots, graphene quantum dots) along with their unique characteristics showed sensitive thermal properties which make them excellent candidates for thermometric behavior at the nano-scale domain. Here, definitions, advantages, and mechanisms of the thermal-sensing behavior of carbon dots are reviewed. Finally, future perspectives of this new class of thermo-materials are presented.

## Nanothermometry

What is meant by “nano-thermometry” is using nano-scale thermosensitive materials to give temperature information about the local environment of the nano or micro-scale region [21, 22]. Nanoparticle-based thermal probes have great capacity in a broad spectrum of sensing applications, and numerous progressive so-called “nanothermometers” have lately been reported. Moreover, different kinds of conventional nanomaterials have been reported to have thermosensitive luminescent properties, such as polymers [23, 24], nanocrystals [25], rare-earth-doped nanoparticles [26, 27], and metal nanoparticles [28].

Thus, nanothermosensors are non-invasive, non-contact-mode, accurate thermometers working at the nanoscale with high-sensitivity resolution [9]. Thermal sensing utilizing nanomaterials can be achieved by manipulating their optical properties. Fluorescence nanothermometry can be classified into quite a few classes relying on the precise parameter from which the thermal measurement is derived involving intensity of the signal, shape of the band, fluorescence lifetimes, band-shift, polarization of the excitation wavelength, and spectral shift. In the first case, fluorescence changes with different temperatures and may be detected as an absolute increase (or decrease) in signal [9, 29–31].

Contact-less luminescence nanothermometry, which uses luminescent nanomaterials with temperature-dependent emissions, is predominantly appropriate for biological applications [32, 33]. These luminescent nanomaterials include fluorescent polymers [24], metallic nanoparticles [34], rare-earth-doped nanoparticles [35], and nanodiamonds [36] which have a thermo-sensitive property in the physiological range. These pioneering works were capable to deliver the average temperature for individual cells. Temperature-dependent luminescent probes based on organic dyes (e.g., Rhodamine 6G) and polymers (e.g., poly(*N*-isopropyl acrylamide)) generally display poor photostability and pronounced cross-sensitivity to oxygen, which is undesirable for live-cell work [8]. Additionally, there is a strong pH dependence on the lifetime of the fluorophore, which makes it difficult to use without the precise controlling of the pH of the environment being surveyed [37].

Another class of nanothermometers based on both pure and doped semiconductor nanocrystals has been reported, with most prominent candidates such as CdSe, ZnS, InP, or PbSe [19, 38–40]. Semiconductor quantum dots (SQDs) are a candidate for nanoscale thermometers, considering they have high quantum yield, a long lifetime before photobleaching, and adequate biocompatibility after proper surface modification. Furthermore, they can be easily conjugated to proteins and DNA for sensing and imaging [41]. The exceptional challenge faced by this type of luminescent thermometers is the associated recognition of brightness, photostability, sensitivity, and precision at *T* = 20–40 °C when probing subcellular microenvironments. SQDs have been extensively reviewed in terms of synthesis, physicochemical properties, luminescence, as well as their potential applications. Here, we direct the attention of the reader to these numerous remarkable reviews [42–46]. Compared to organic dyes, SQDs exhibit superior brightness for detection, a wider excitation profile for multiplexing, and better photostability for long-term studies. Furthermore, SQDs as temperature sensors are resistant to pH and other environmental variations that are expected to be prevalent inside a cell [47].

Generally, in SQDs due to a combination of different processes, an increase in temperature produces a decrease in the fluorescence intensity (quenching), which is accompanied by a spectral shift. This shift can be assumed to be linear in the biophysical range. The magnitude of both effects (luminescence quenching and spectral shift) depends strongly on the material constituting the QDs and on their size [48].

Each group of luminescent nanomaterials has limitations of use along with their advantages. As described above, SQDs are favored more than fluorescent polymers and organic dyes. SQDs are good in terms of photostability, quantum efficiency, and tunable fluorescence, but QDs cannot be used to trace a single molecule for long-term monitoring because of their intrinsic blinking [49]. Moreover, the main pitfall of QDs is their toxicity, which is due to their heavy metal content, including metals such as cadmium; this limits their biological and environmental applications. Besides, the availability of precursor elements in nature is relatively low and therefore SQDs are considered to be expensive [50].

## Carbon Dots as Nanothermometers

In order to overcome problems that arose from non-carbon-based nanothermometers (as we explained in the previous section), carbon-based nanomaterials have been prepared and displayed unique properties, such as low toxicity, facile preparation, low-cost precursor, photostability, and biocompatibility. These carbon-based nanomaterials showed sensitive thermal sensing properties. Additionally, the improvement of metal-free nanoparticles is important and urgent due to the environmental hazard for biological applications for such toxic materials [51, 52]. Among the family of carbon-based nanomaterials, fluorescent nanodiamonds have been first reported as nanothermometers [53]. Fluorescent nanodiamonds possess intrinsic biocompatibility resulting from their chemically robust and inert surface [54]. Other nanodiamonds have recently been used for intracellular thermal sensing with sub-degree accuracy [55, 56]. The thermal sensitivity of these nanodiamonds is based on the so-called nitrogen-vacancy color centers, which are point defects consisting of a nitrogen atom replacing a lattice carbon atom and a nearby vacant lattice site [48, 57]. The nitrogen-vacancy center of fluorescent nanodiamonds is studied extensively and has been well-characterized for its photophysics, as well as its use in biological applications [58]. The functioning principle of nitrogen-vacancy-based thermometry depends on the accurate measurement of this color center, which can be optically detected with high spatial resolution [30, 59]. However, low fluorescence efficiency and poor controllability impede the application of fluorescent nanodiamonds tremendously [36].

One of the newer class of carbon-based nanomaterials family is highly luminescent carbon dots (CDs), that have exceptional bright photoluminescence, photochemical stability, water-solubility, great biocompatibility, and non-toxicity [60–62]. CDs are zero-dimensional and sphere-shaped nanoparticles possessing diameters less than 10 nm [63, 64]. Various approaches have been applied for preparing diverse types of CDs, e.g., laser ablation [65], solvothermal [66], hydrothermal synthesis [67], microwave-assisted [68], arc discharge [69], acidic oxidation [70], and more chemical and physical approaches [71, 72]. Additionally, CDs display a desirable prospect for various applications, such as biological imaging [73], chemical and biosensing [74–77], targeted drug delivery [78], pharmaceutical analysis [79], and catalysis [80, 81]. Carbon dots have been extensively reviewed in term of synthesis, physicochemical properties, as well as their potential applications. Here, we refer the reader to the numerous good reviews on carbon dots [72, 82–88].

In recent years, fluorescent CDs as temperature sensors have attracted much attention from researchers. In principle, some requirement is needed for the effective temperature measurement, like carbon nanodots should exhibit appreciable variation in their photoluminescence over the range of relevant temperatures [89]. The photostability, pH stability, and shelf-life are other requirements that should be taken into consideration for practical application.

CDs are promising alternatives for conventional semiconductor quantum dots (SQDs). Compared with QDs, CDs show many outstanding advantages such as low cost, low toxicity, and their unique robust optical/chemical properties [90] Additionally, CDs show very less photobleaching. Compared to other fluorescent raw materials, CDs are produced from inexpensive carbon sources that are abundant in nature [91]. Furthermore, there are several simple methods to modify and functionalize the surface state of CDs, which allow tailoring the solubility, stability, physicochemical properties, and quantum yields of CDs according to their experimental requirements [49, 92, 93].

In literature, there is a scarce number of reported articles of carbon dots with temperature-dependent fluorescence and are shown in Table 1.

**Table 1:** Temperature-dependent fluorescent carbon dots reported up to now

Carbon precursor	Synthesis method	Thermometer nanomaterial	Linear range (°C)	Sensitivity	Reference
Glacial acetic acid	Self-promoted and self-controlled	CDs	–	–	[51]
Glucose	Hydrothermal	Passivated CDs	15–60	–	[1]
C3N4	Reflux	Nitrogen-doped CDs	20–80	0.85% °C^−1^	[94]
l-cysteine	Hydrothermal	Nitrogen and sulfur co-doped CDs	10–90	–	[95]
Acrylic acid, Methionine	Hydrothermal	Nitrogen and sulfur co-doped CDs	25–75	–	[92]
Citric acid	Hydrothermal	Nitrogen and sulfur co-doped CDs	-	1.79% K^−1^	[89]
C_3_N_3_S_3_	Reflux	Nitrogen and sulfur co-doped CDs	20–80	–	[96]
Graphite	Laser ablation	CDs	5–85	1.48% °C^−1^	[14]
D-glucose	Hydrothermal	Nitrogen and sulfur co-doped CDs	5–75	–	[97]
Citric acid	Hydrothermal	Nitrogen and sulfur co-doped CDs	5-75	0.41% °C^−1^	[2]
Citric acid	Hydrothermal	CDs	25–95	–	[98]
Dextrose	Hydrothermal	Nitrogen-doped CDs	25–95	–	[99]
Formamide, glutathione	Microwave-assisted	CDs	5–60	3.71% C^−1^	[29]
C_3_N_3_S_3_	Hydrothermal	Nitrogen, sulfur and iodine co-doped CDs	10–8	–	[100]
Cetylpyridinium chloride	Hydrothermal	Nitrogen and chlorine co-doped CDs	20–80	–	[101]
Trisodium citrate	Hydrothermal	Nitrogen and sulfur co-doped CDs	283–343 (K)	0.64% K^−1^	[102]
Manganese (III) acetylacetonate	Hydrothermal	Manganese oxide doped CDs	10–60	–	[103]
Trisodium citrate	Hydrothermal	Nitrogen and boron co-doped CDs	0–90	1.8% °C^−1^	[77]

**Fig. 2:**
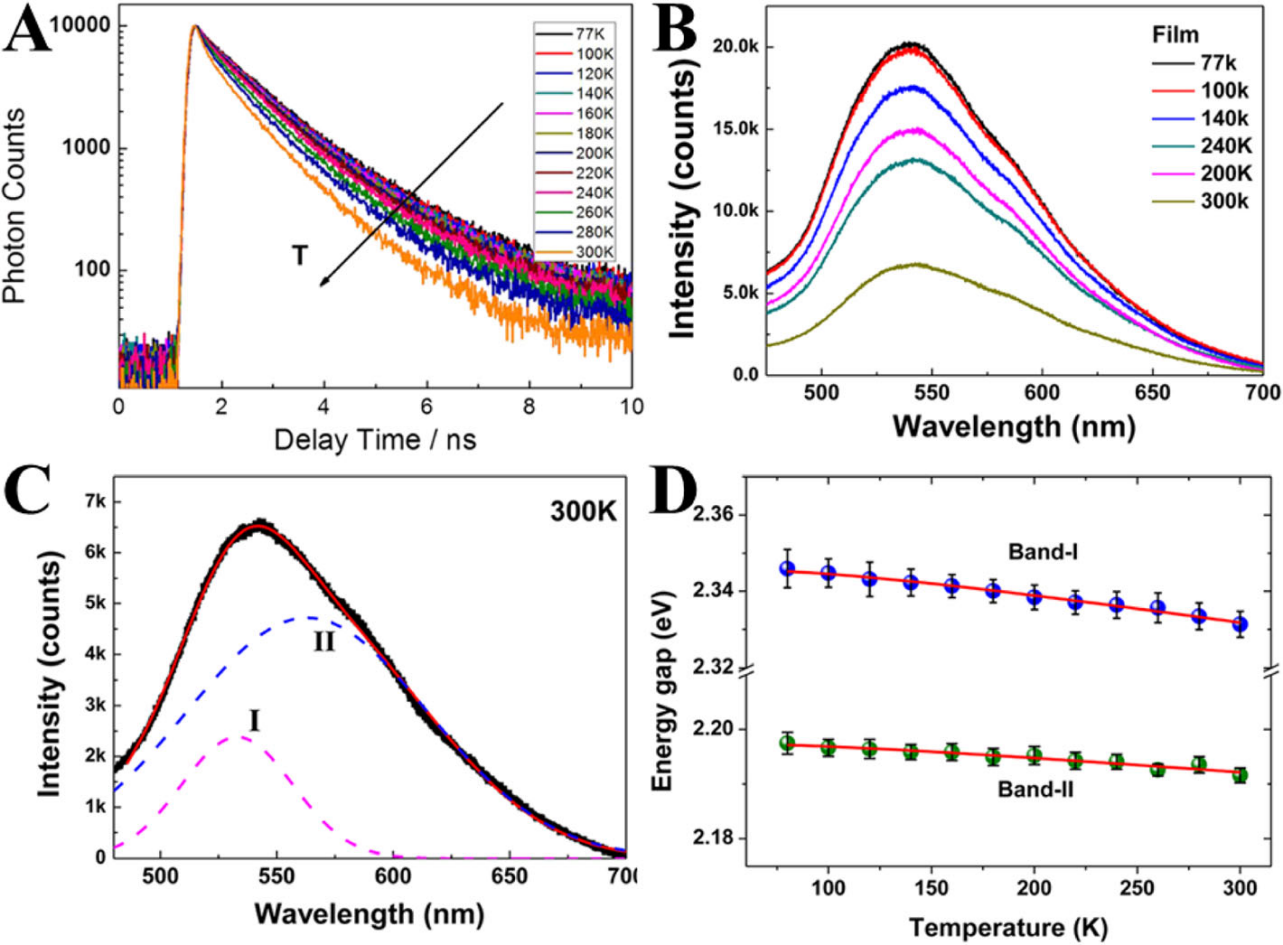
**a** Time-resolved photoluminescence measurements as a function of temperature. **b** Fluorescence spectra of CD film as a function of temperature. c Fluorescence spectra at 300 K fitted by a two-Gaussian function. **d** The bandwidth of fluorescence as a function of temperature. (Reproduced with permission from reference [51])

Yu et al. [51] in 2012 were the first to investigate the temperature-dependent fluorescence in carbon nanodots and compared them to semiconductors and metal-based nanoparticles. They depended upon measuring temperature-dependent photoluminescence lifetimes via the time-correlated single-photon counting technique (TCSPC). The photoluminescence relaxation dynamics become faster at higher temperatures (Fig. 2a), which can be ascribed to the nonradiative decay processes. The measurement of the fluorescence spectra of the CD film as a function of temperature from cryogenic to room temperature was performed (Fig. 2b). With an increase in temperature, the intensity of fluorescence displays a repetitive decrease.

The PL spectrum exhibits asymmetric peaks, therefore the PL spectra at each temperature could be well fitted by a two-Gaussian function shown in Fig. 2c. The high energy band, band I; the low energy band, band II (Fig. 2d).

Furthermore, the energy gap (bandwidth) of fluorescence was inspected as a function of temperature (Fig. 2d). The total bandwidth is pronounced by temperature-independent (electron-electron scattering), and temperature-dependent (electron-phonon and surface/defect scattering). The bandwidth of band I and band II is temperature-independent, which specifies that electron-electron scattering dominates in the CDs.

Therefore, the weak temperature effect in CDs is in agreement with the fact that the principal interaction mechanism involves electron-electron interactions rather than electron-phonon coupling. Additionally, a broad PL band (> 100 nm) usually observed even at very low temperature (77 K) is referred to strong electron-electron interaction (Fig. 2c). This result is similar to metallic nanoclusters and different from semiconductor QDs. Hence, Yu and coworkers speculated that the π-electrons in CDs can act similarly as free-electrons in the metallic nanoclusters.

**Fig. 3.**
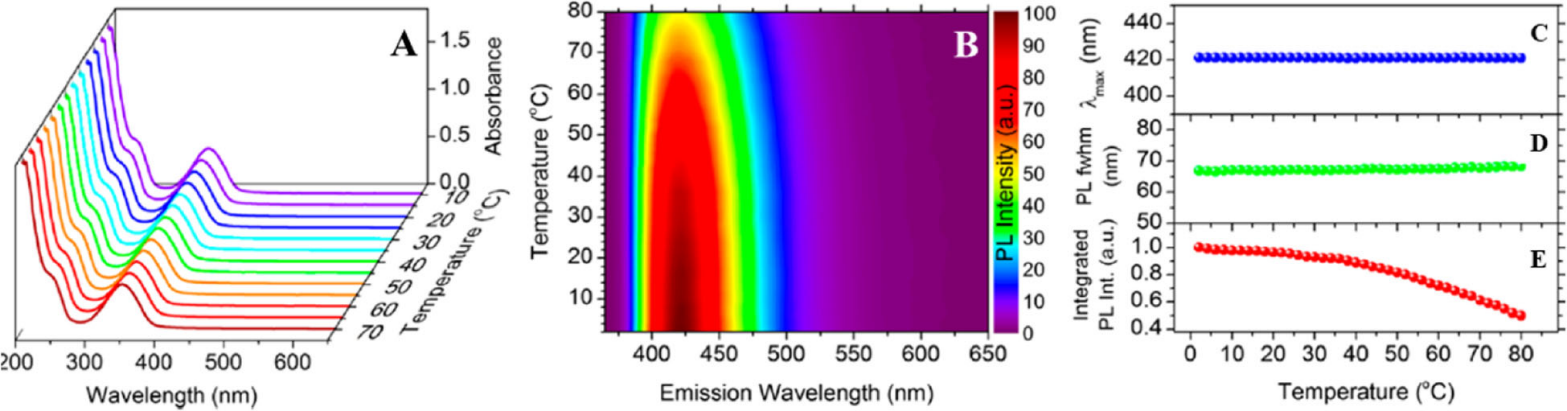
**a** Temperature-dependent absorption taken in the temperature range from 10 to 70 °C. **b** Normalized color plot of the temperature-dependent PL emission at temperatures between 2 and 80 °C with a step size of 2 °C and excitation at 355 nm. Corresponding temperature-dependent changes in the PL peak maximum λmax (**c**), PL fwhm (**d**), and integrated PL intensity (**e**). (Reproduced from reference [89])

Kalytchuk et al. [89] synthesized highly luminescent water-soluble N, S-CDs by one-step hydrothermal treatment of citric acid and l-cysteine. They collected steady-state absorption spectra at a wide range of temperatures to characterize the temperature dependence PL properties of the CDs. The absorption spectra of N, S-CDs dispersed in water at temperatures between 10 and 70 °C (increasing by 5 °C per step) are shown in Fig. 3a. Unlike in semiconductor nanocrystals, the position and the intensity of the absorption band did not change with the temperature. Similar results were previously reported for CDs synthesized by hydrothermal treatment of glucose in the presence of glutathione [1].

In their work, they showed a color plot of the PL spectra of N, S-CDs acquired at temperatures ranging from 2 to 80 °C with a step of 2 °C (Fig. 3b). Increasing the temperature reduced the PL emission intensity by approximately a factor of 2 without any detectable shift of the PL emissions [89]. The position of the PL emission maximum, the PL full width at half- maximum (fwhm), and the integrated PL intensity were quantitatively determined at the studied temperature, condensing the results in Fig 3c–e, respectively.

The PL peak position of N, S-CDs shows weak temperature dependence, unlike most of the semiconductor nanocrystals, whose bandgap changes with temperature, inducing the PL emission shift. Moreover, their PL fwhm exhibits only insignificant broadening (1.4 ± 1 nm) over the same temperature range (Fig. 3d), indicating that the PL peak of the N, S-CDs shows negligible thermal broadening. To characterize the nonradiative relaxation processes occurring in the CDs, they analyzed the quenching of the integrated PL intensity as a function of the temperature. A plot of the temperature-dependent integrated PL intensity for N, S-CDs is shown in Fig. 3e, with the values normalized to the intensity at 2 °C, revealing that the intensity decreases monotonically over the studied temperature range, with that at 80 °C being approximately half that at 2 °C. Based on these results, the activation energy of thermal quenching for the CDs at temperatures between 2 and 80 °C was estimated to be 17.0 ± 0.7 meV using the Arrhenius formula for N, S-CDs, which is close to the value reported by Yu et al. [51].

**Fig. 4:**
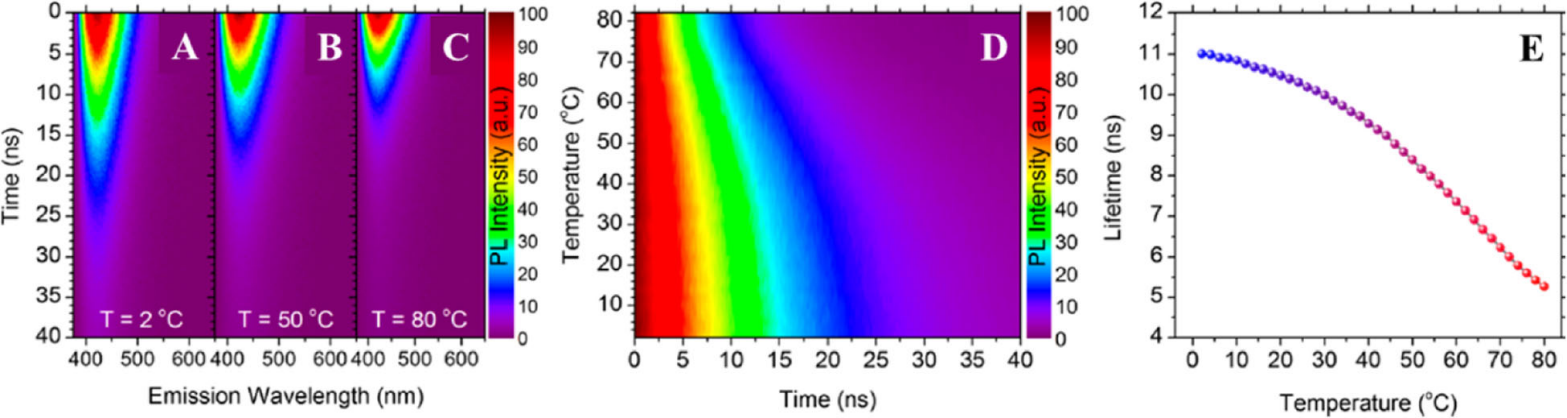
Time-resolved temperature-dependent PL emission of CDs. Normalized color plots showing time-resolved PL emission maps for CDs at **a** 2 °C, **b** 50 °C, and **c** 80 °C. **d** The normalized color plot of time-resolved PL intensity at the PL emission maximum (λem = 421 nm) at temperatures between 2 and 80 °C. **e** Extracted PL lifetimes plotted as a function of temperature in the range of 2–80 °C. (Reproduced from reference [89])

Besides, Kalytchuk et al. [89] examined CD’s emission dynamics at different temperatures. Figure 4a–c exhibits the strong temperature dependence of their time-resolved PL emission, showing time-resolved emission spectroscopy data for three different temperatures. Transient PL emission maps of CDs were acquired in the spectral region between 375 and 650 nm at 2, 50, and 80 °C, as shown in Fig. 4a–c. There is a clear decline in PL decay as the temperature increases, suggesting that the CDs have satisfactory properties for PL lifetime-based temperature sensing. It is important to note that spectrally uniform single-exponential decay was observed across the dots’ emission profiles at all studied temperatures, indicating that recombination occurs via very similar and highly emissive channels across the entire CD ensemble. The CDs’ PL dynamics are arguably their most promising quality relating to temperature sensing applications. Specifically, the temperature sensitivity of CDs makes them PL lifetime nanothermometers.

The variation of the CDs’ PL lifetime at temperatures between 2 and 80 °C have been investigated thoroughly; Fig. 4d, e shows time-resolved PL data collected at the dots’ PL emission maximum as a function of the temperature. A color plot of transient PL across the studied temperature range is presented in Fig. 4d, showing that increasing the temperature leads to a monotonic shortening of the apparent PL decay. All of the recorded decay curves were fitted using a single-exponential function. Data on the extracted lifetimes are presented in Fig. 4e. As the temperature increases from 2 to 80 °C, the PL lifetime decreases monotonically from 11.0 to 5.3 ns. The temperature range over which this PL lifetime sensitivity has been demonstrated (2–80 °C) covers both the physiologically relevant temperature range and the typical operating temperatures of many electronic devices. The absolute pseudo-linear sensitivity of this PL lifetime CD-based thermal probe is 0.08 ns K^−1^, and its maximum relative sensitivity is 1.79% K^−1^ at 62 °C. The single-exponential fit of the PL decay of CD-based luminescent nanoprobe across the studied temperature range yields a single parameter, the PL lifetime (τ), which can be directly converted to temperature units using a calibration curve. This is an important advantage over typical semiconductor quantum dots, which exhibit multi-exponential decay that limits their usefulness in applications involving PL lifetime measurements.

They also studied the temperature dependence of CDs PL lifetime in phosphate-buffered saline (PBS) and Dulbecco’s modified Eagle’s medium (DMEM) and showed similar behavior.

**Fig. 5:**
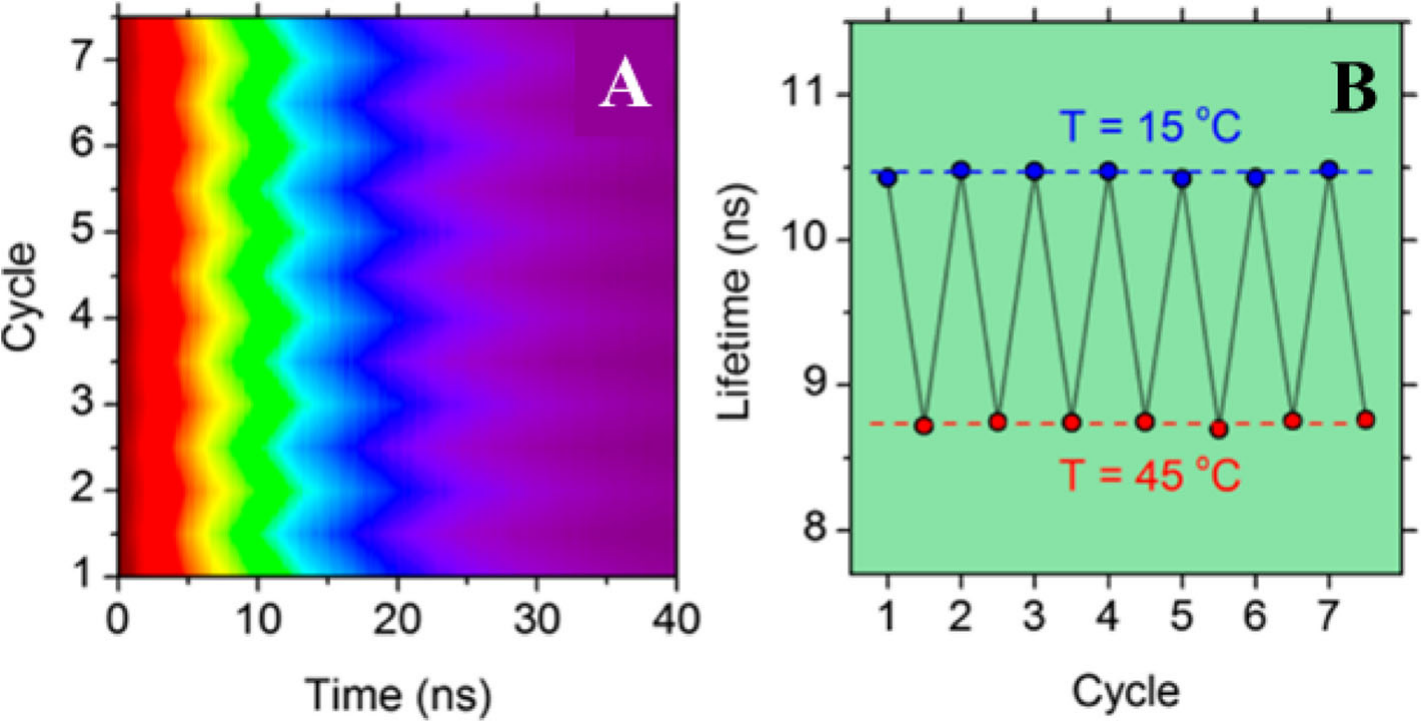
**a** Normalized color plot of PL decay reversibility over seven sequential cycles of heating and cooling. **b** Corresponding thermal stability of PL lifetimes over seven cycles of heating and cooling between 15 and 40 °C. (Reproduced from reference [89])

To demonstrate the reusability of CD-based luminescence thermometers, the PL decay curves of selected samples were measured over seven successive cycles of heating and cooling at temperatures between 15 and 45 °C (Fig. 5a).

In each measurement cycle, the PL decay was measured after 5-min thermal equilibration. No thermal hysteresis was observed during the heating and cooling cycles, and the resulting PL lifetime variation is plotted as a function of time in (Fig. 5b), demonstrating that the PL lifetime of the CDs exhibits excellent thermal stability.

**Fig. 6.**
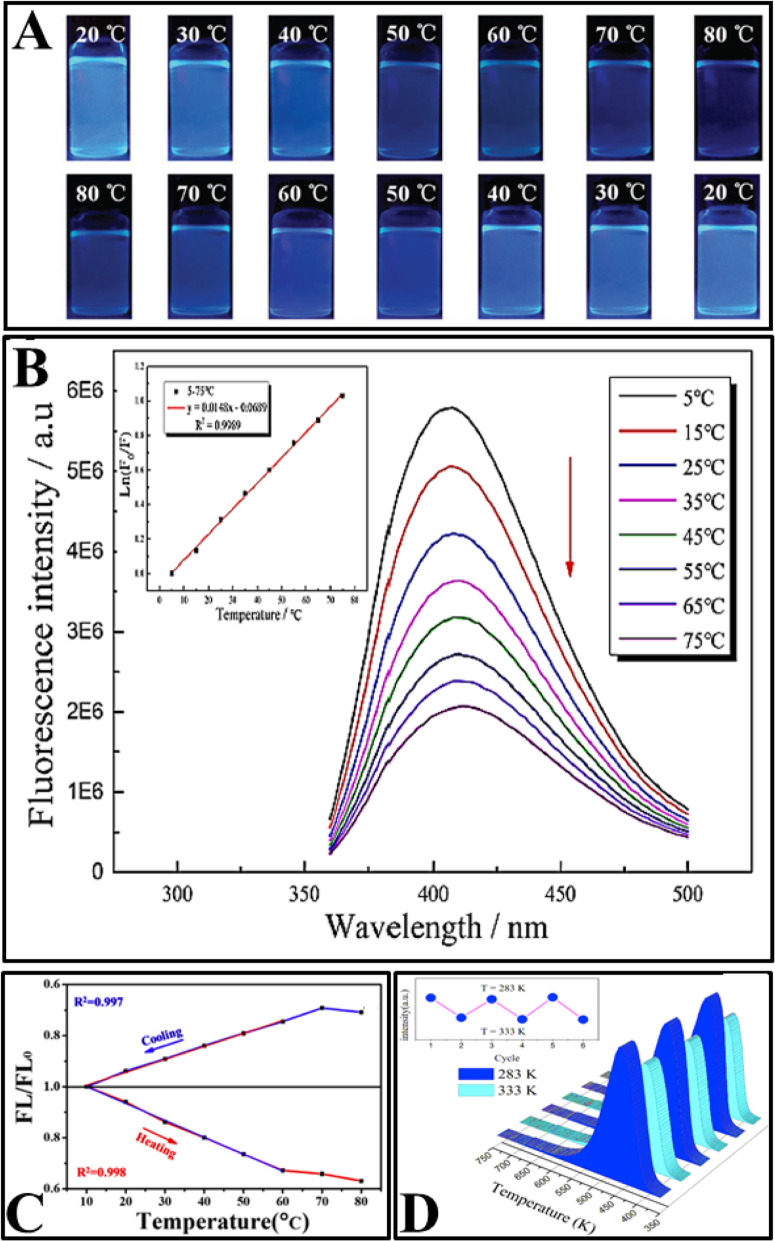
**a** Digital photographs of N, S-CDs under UV light (365 nm) excitation at different temperatures during heating (upper) and cooling processes (lower). (Reproduced from reference [96]). **b** Fluorescence emission spectra of N, S co-doped CDs measured in the range of 5–75 °C (top to bottom) when excited at 340 nm, inset: the corresponding linear regression of the temperature versus Ln (*F*/*F*_0_). (Reproduced from reference [97]). **c** FL/FL_0_-temperature plots of MnOx-CDs during cooling and heating processes. (Reproduced from reference [103]). **d** Reversible temperature-dependence of the PL of the CDs solution. (Reproduced from reference [102])

Later, several CDs with temperature-dependent emission were prepared using a variety of synthesis methods, such as hydrothermal and solvothermal treatment [2, 77, 92, 95, 97–103], heat reflux [94, 96], and laser ablation [14] as shown in Table 1. The prepared CDs showed linear temperature-dependent fluorescence at the physiological ranges (shown in Table 1). The fluorescence intensity of the CDs decreased with increasing temperature. Furthermore, the reversibility and restorability of the fluorescence intensity have been studied in all articles. Figure 6 shows some common properties of CDs studied in various articles.

**Fig. 7:**
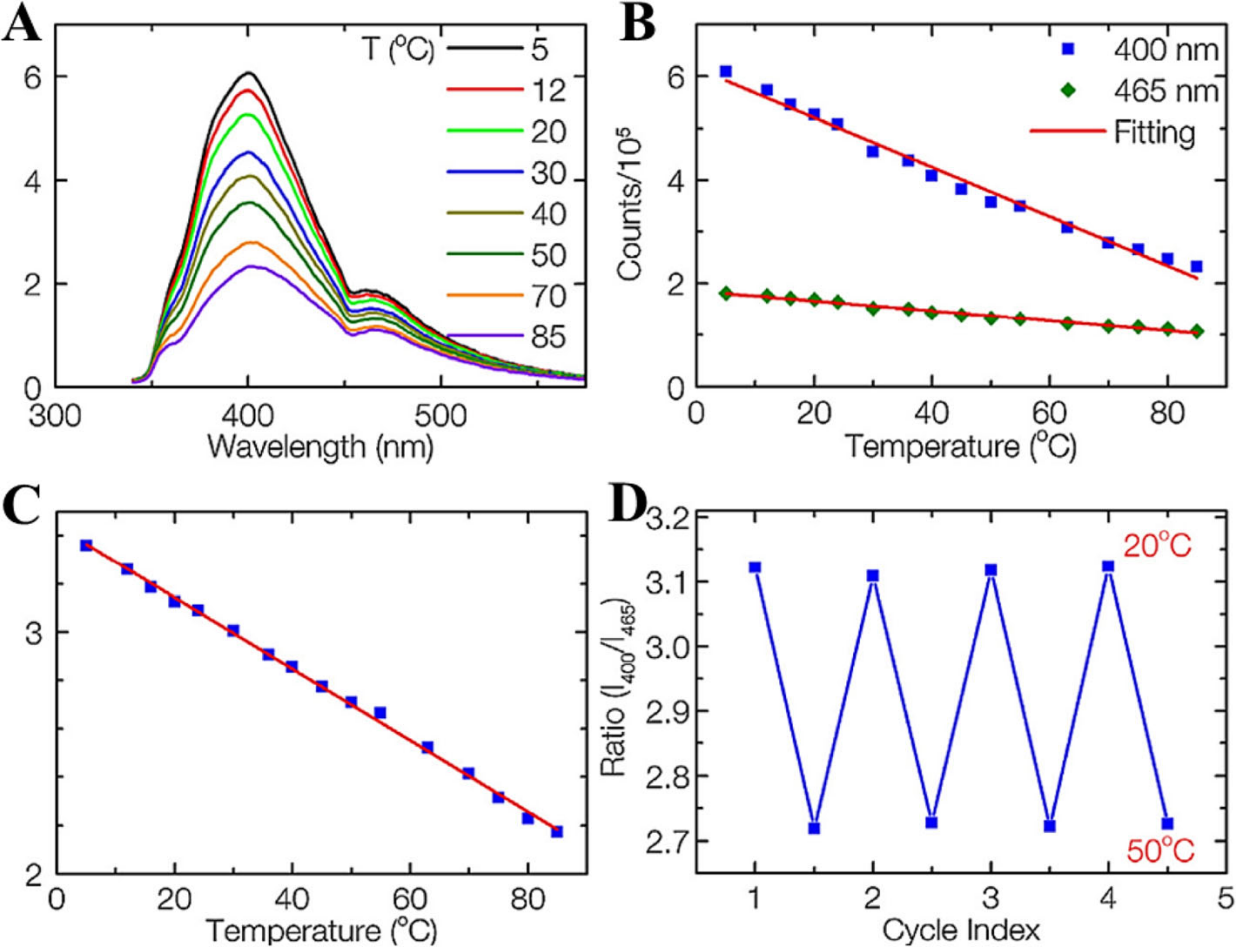
**a** Emission spectra of CDs recorded from 5 to 85 °C, excited at 320 nm. **b** Fluorescence intensities of the 400 and 465 nm peaks versus temperature. **c** The ratio of the 400 nm over the 465 nm peaks as a function of temperature. **d** Temperature reversibility study of CDs between 20 and 50 °C. (Reproduced from reference [14])

Nguyen et al. [14] synthesized carbon dots (CDs) using femtosecond laser ablation of graphite powder in ethylene-diamine. Abundant functional groups were formed on the surface which forms multiple surface states on the surface site and results in the multi-emission of CDs. They investigated the fluorescence-dependent temperature sensitivity of CDs using steady-state fluorescence spectra. The temperature-dependent emission spectra of the CDs at 320 nm excitation are shown in Fig. 7a. Both fluorescence intensities of 400 and 465 nm peaks gradually decrease with the increase of temperature due to the thermal activation of nonradiative-decay pathways. The peak intensities change linearly with temperature ranging from 5 to 85 °C (Fig. 7b). Temperature-sensitive CDs showed a fluorescence-intensities change of 3.3 and 2.1% per °C for 400 and 465 nm peaks, respectively. This indicates that the CDs can be used as a conventional intensity-based temperature sensor with high sensitivity.

Remarkably, the unique multi-emission property makes the CDs promising fluorophores for ratiometric fluorescent temperature sensors. The ratio of the two fluorescence intensities at 400 and 465 nm (320 nm excitation) versus temperature is shown in Fig. 5c. There is a very good linear relationship between the intensity ratio and temperature in a wide temperature range from 5 to 85 °C (*R*^2^ = 0.998). Thermal linearity is advantageous since it makes the correlation between the peak-intensity ratio and temperature straightforward and meanwhile provides a constant thermal sensitivity along with the entire dynamic range. The temperature-sensitivity of CDs is determined to be 1.48% °C^−1^, which is comparable with that of other materials. It should be noted that the temperature response range of CDs is much wider than those of other reports on dual-emission temperature sensors and covers both the physiological temperature for biology studies and the working temperature for many electronic devices. Besides 320 nm excitation, the CDs also work at other excitation wavelengths, such as 340 and 365 nm, with the same sensitivity. Thus, the CDs can be utilized for temperature sensing in many practical applications by selected different working wavelengths.

They have shown that the ratiometric temperature sensor was reversible between 20 and 50 °C, four cycles and photostable (when the intensity of the power source changed) as shown in Fig. 7d. This result suggests that the CDs sensing system is stable and robust with any changes in sample concentration, excitation, or detection efficiency.

**Fig. 8.**
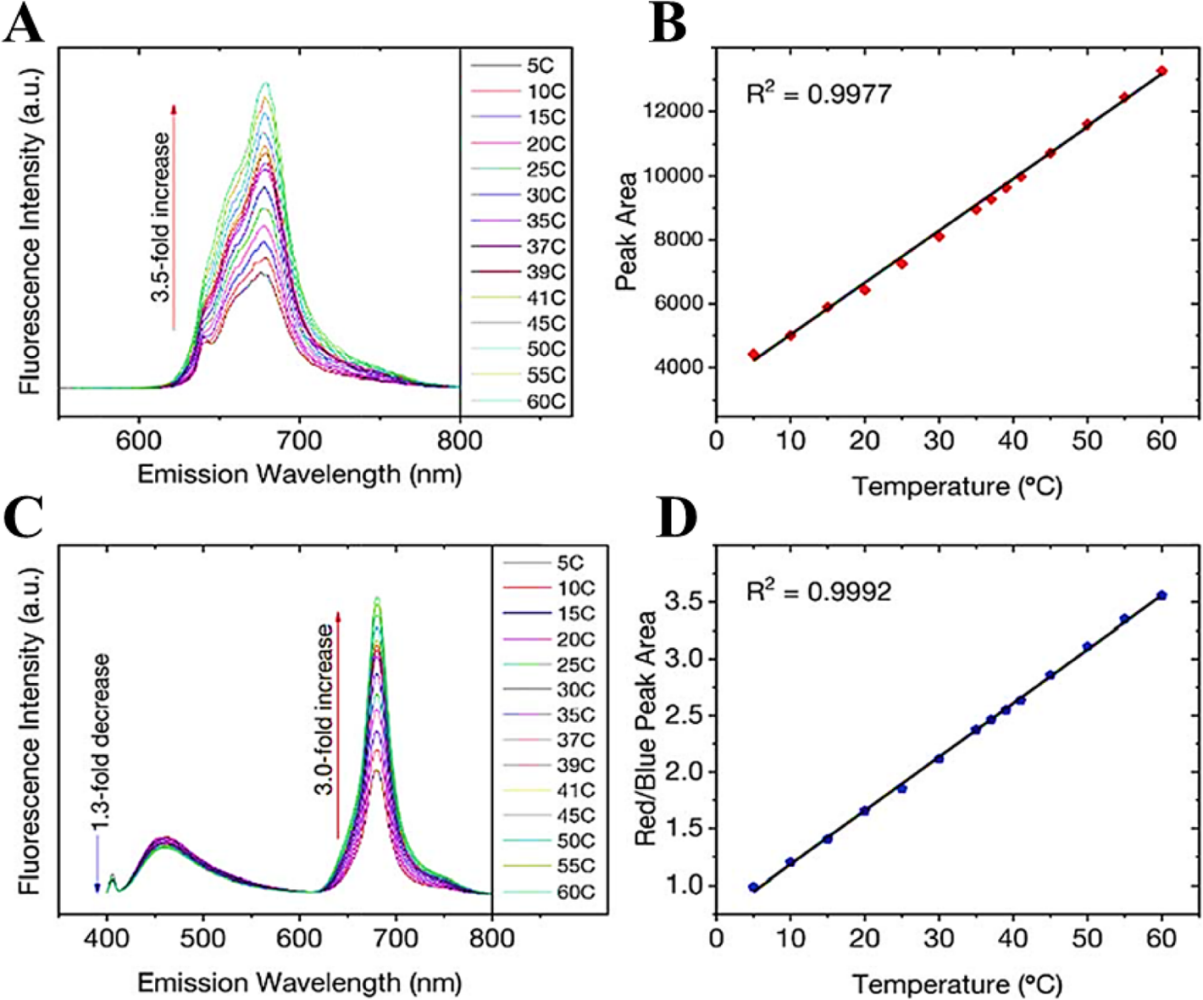
**a** Excitation at 640 nm yields a 3.5-fold increase in fluorescence intensity and the corresponding integrated area is plotted in **b** showing a linear response over the range of 5–60 °C. **c** Changes in the fluorescence spectra of the CDs (λ_ex_ = 405 nm) as a function of temperature over the entire range. A 1.3-fold decrease is noted for the blue fluorescence in contrast to the 3-fold increase for the red counterpart. **d** The ratio of the integrated areas of the red and blue fluorescence components are plotted as a function of temperature showing a linear increase over the entire temperature range. (Reproduced from reference [29])

Increasing temperature is not always accompanied by PL quenching; however, it could show enhancement of the PL as well. Macairan et al. [29] showed the PL enhancement of dual-fluorescent carbon dots with increasing temperature. They prepared biocompatible dual-fluorescing carbon dots CDs in a one-step microwave assisted-reaction using formamide and glutathione. They found that following excitation at 640 nm, the fluorescence intensity and PL integrated area increase over the range of 5–60 C by a factor of 3.5 observed over the entire analysis range and the temperature (Fig. 8a). As shown in Fig. 8b, a linear response (*R*^2^ = 0.999) is observed over the entire analysis range and the temperature sensitivity was determined to be as high as 3.71% C^−1^.

The temperature-dependent fluorescence was also studied following excitation at 405 nm. Interestingly, the blue and red fluorescence bands are not equally sensitive to the change in temperature. With increasing temperature, the fluorescence intensity (and the corresponding integrated area under the curve) of the blue component shows a very slight decrease in contrast to the red component, which significantly increases (Fig. 8c). These observations are noted over the range of 5–60 °C where the blue emission decreases by a factor of 1.3 in contrast to the red emission, which increases by a factor of 3.0.

As shown in Fig. 8d, the ratio of red to blue fluorescence increases with temperature, and a highly linear response is triplicate on 3 unique samples and the linear plot reflects the observed with an *R*^2^ = 0.998. These analyses were repeated in an average of these measurements, which have small deviations at each temperature. The thermal sensitivity of the CDs, over the entire temperature range, varied from 1.33 to 4.81% °C^−1^, which is an improvement over previously reported carbon dot nano-thermometry systems and other dual-emitting nanomaterials such as quantum dots and metal-organic frameworks-dye composites. The thermal resolution of the CDs was calculated to be 0.048 K^−1^ indicating that it is indeed possible to measure small thermal changes.

**Fig. 9:**
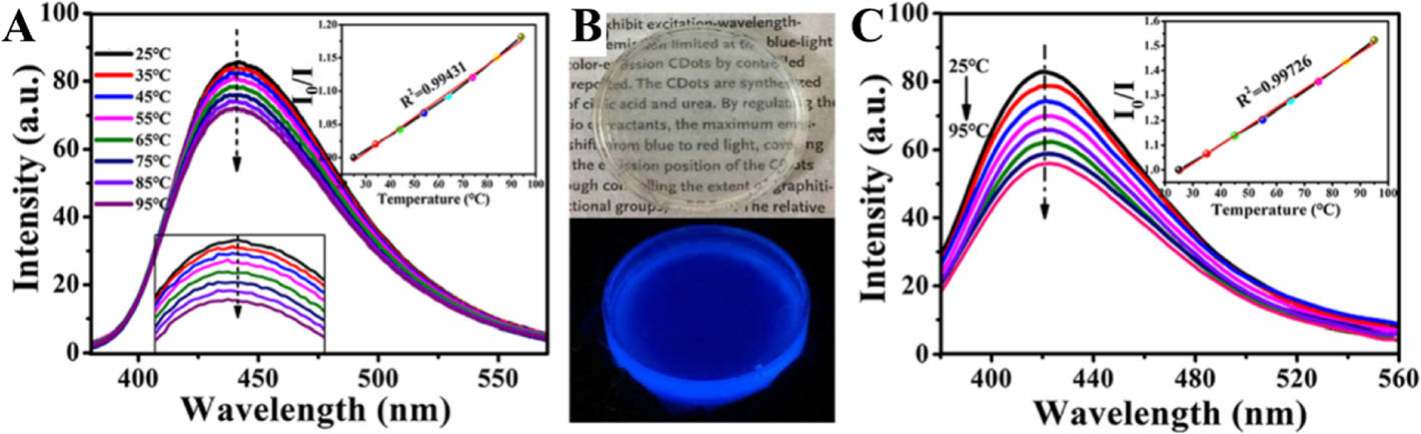
**a** Temperature dependence of the CD emission. **b** CD/epoxy composites. **c** Temperature dependence of the emission of the CD/epoxy composites. (Reproduced from reference [98])

Zhang et al. [98] synthesized CDs that have temperature-responsive characteristics in the range of 25–95 °C, and they have excellent sensitivity and remarkable reversibility/recoverability (Fig. 9a). CD/epoxy composites were further prepared by uniformly doping CDs into an epoxy resin. First, 5 μg of the CDs were dissolved in 50 μL of triethylenetetramine (TETA). Then, 350 μL epoxy resin was added to the mixed solution and fully mixed by high-speed stirring. The resulting composite showed significantly enhanced temperature response.

Epoxy resin is a common thermosetting resin and is widely used to package LED chips. Figure 9b shows optical micrographs of CD/epoxy composite discs of approximately 2 cm in diameter and 8–10 mm in thickness. The cured CD/epoxy composites are transparent, and their fluorescence emission spectra are shown in Fig. 9c. The emission peak of the CD/epoxy composite is blue-shifted by approximately 10 nm compared to that of the CDs’ solution. Notably, the temperature response of the composite is significantly improved. In the temperature range of 25–95 °C, the fluorescence intensity decreases by 35% with increasing temperature, which is more than twice that of the solution state, and the linear results are more stable. The linear equation satisfies *I*_0_/*I* = 0.0074 [°C] + 0.80454 (*R*^2^ = 0.99724), where *I*_0_ and *I* are the fluorescence intensity of the CD/epoxy composite before and after the temperature rise, and the excitation wavelength is 360 nm. The blue shift of the emission peak and the enhancement of the temperature response characteristics may be due to changes in the dielectric constant of the environment in which the CDs are located. The composite has a wide temperature detection range, and its excellent sensitivity and stability make it suitable for use as a temperature sensor based on a fluorescent nanomaterial in a variety of environments.

## Mechanism of Thermo-sensing

**Fig. 10:**
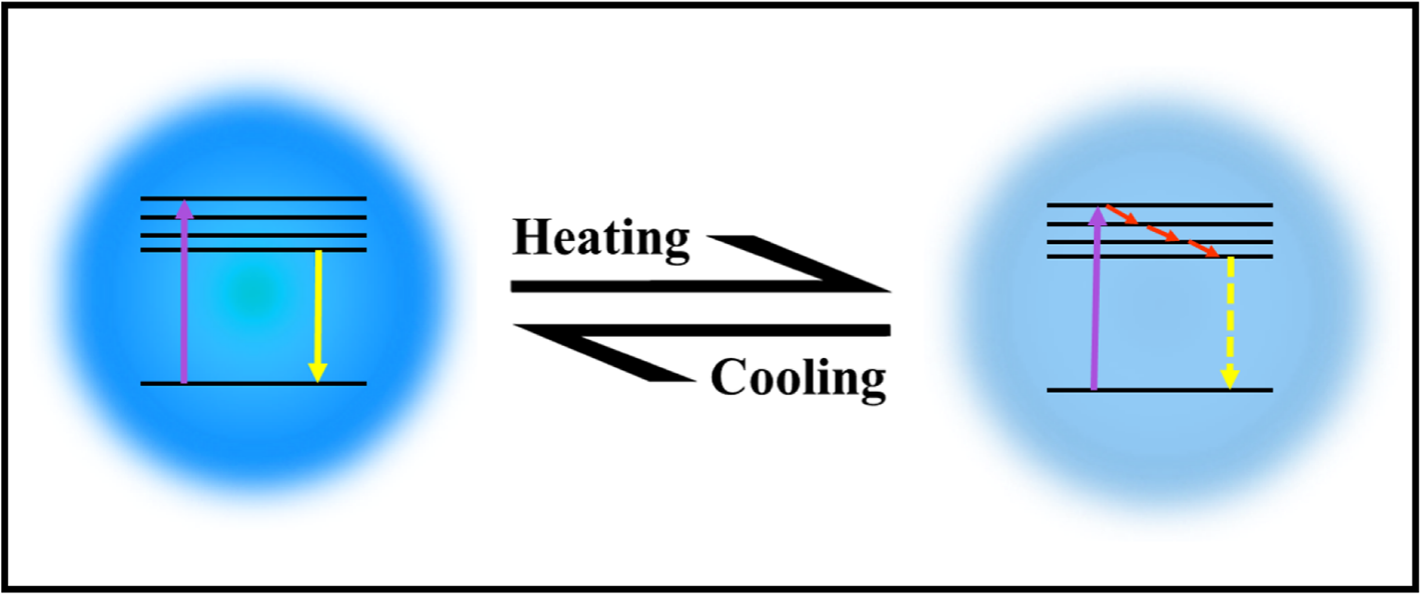
Schematic illustration of CDs responding to temperature changes

Up to now, there is no well-established mechanism for explaining the thermo-sensing behavior of carbon dots. Some reports attribute the mechanism to the thermal activation of non-radiative channels of surface (trap/defect) states. The general picture is that the non-radiative channels were not activated at low temperatures, so the excited electrons could emit photons radiatively. On the contrary, as the temperature increases, more non-radiative channels became activated, and excited electrons got back to the ground state by non-radiative processes, leading to the decreasing fluorescence intensity [2, 95, 99, 100, 103]. The mechanism of CDs emissions with heating/cooling is shown in Fig. 10.

**Fig. 11:**
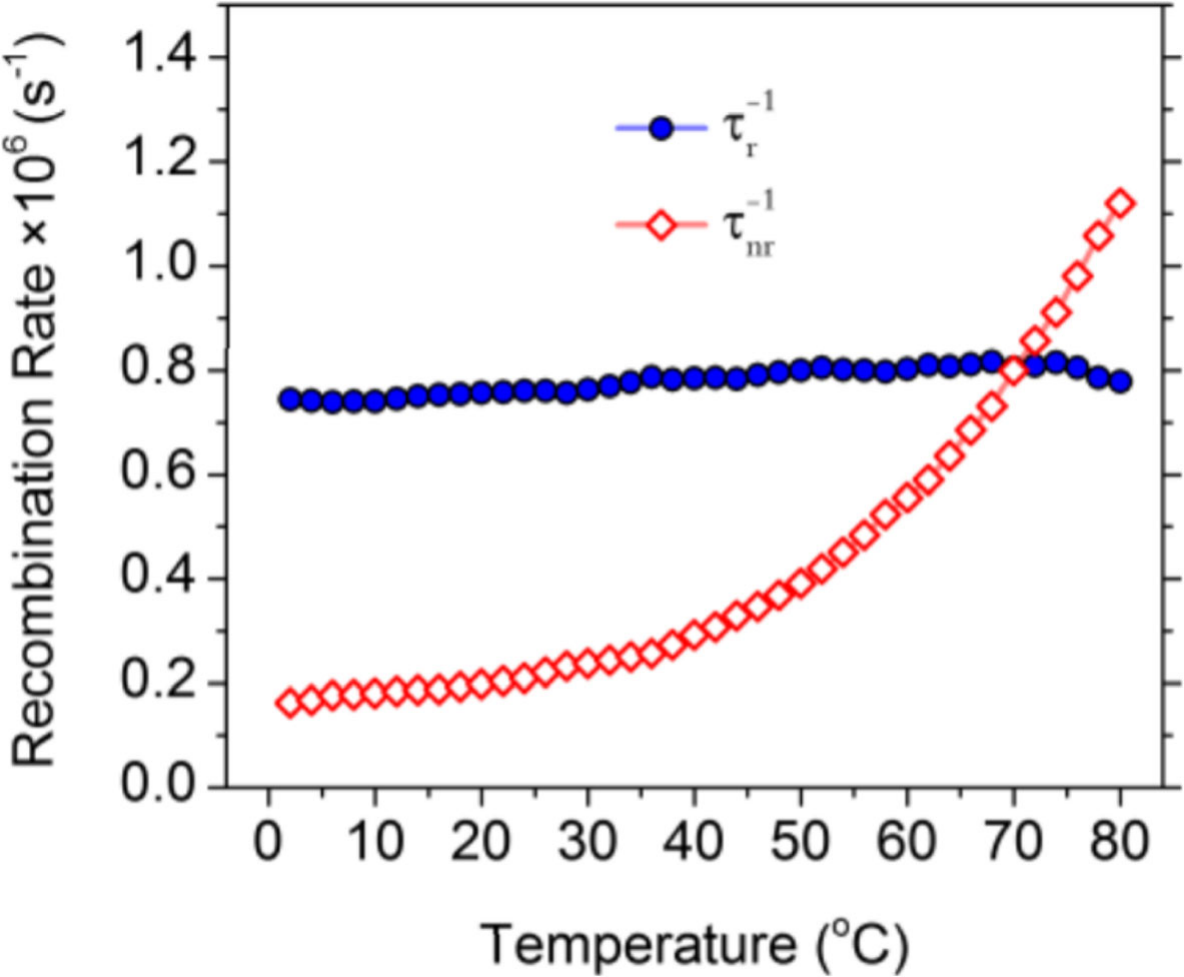
Radiative (solid symbols, blue color) and nonradiative (hollow symbols, red color) recombination rates for CDs plotted against the temperature for temperatures of 2–80 °C. (Reproduced from supporting information of reference [89])

To better understand the thermodynamics of the CD emission processes, Kalytchuk et al. [89] correlated the radiative (${\tau}_r^{-1}$) and nonradiative ( ${\tau}_{nr}^{-1}$) recombination rates of a CD sample with its PL quantum yield. The radiative rate is determined from the PL quantum yield (QY) and the measured recombination rate τ^−1^ as ${\tau}_r^{-1}$ = QY × τ^−1^. The nonradiative relaxation rate ${\tau}_{nr}^{-1}$ is expressed as ${\tau}_{nr}^{-1}$ = τ^−1^ - ${\tau}_r^{-1}$. The PL QY of CDs at various temperatures was calculated from their temperature-dependent absorption and integrated PL intensity together with the PL QY determined at room temperature. Both radiative and nonradiative recombination rates derived from time-resolved PL measurement data are plotted as functions of temperature in Fig. 11. The radiative recombination rate is greater than the correspondent nonradiative rate up to 70 °C and does not vary appreciably at temperatures between 2 and 80 °C, remaining in the range of (0.74–0.82) × 10^6^ s^−1^. In contrast, there is a pronounced (almost 7-fold, from 0.16 × 10^6^ to 1.12 × 10^6^ s^−1^) monotonic increase in the rate of nonradiative recombination by increasing the temperature from 2 to 80 °C. Temperature-dependent crossover of the radiative and nonradiative rates occurs at 70 °C, at which temperature the PL QY is 50%. These results suggest that the temperature activation of PL quenching in their CDs is primarily caused by the activation of nonradiative relaxation channels [89].

**Fig. 12:**
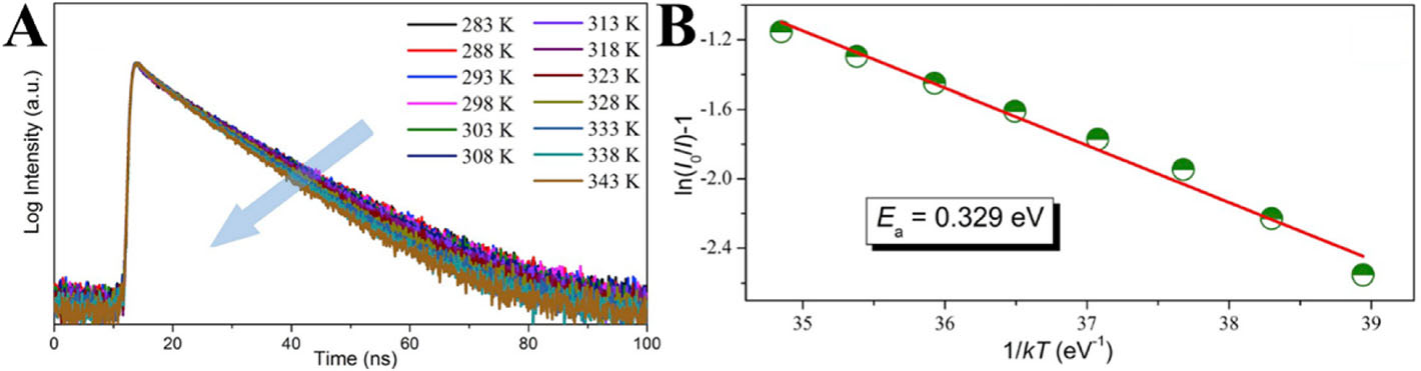
Temperature-dependent decay curves of CDs solution (**a**, λ_ex_ = 350 nm, λ_em_ = 450 nm); the dependence of ln[(*I*_0_/*I*_T_)-1] on 1/kT CDs solution (**b**). (Reproduced from reference [102])

Guo et al. [102] ascribed the thermal-quenching of their prepared carbon dots not just to activation of the nonradiative decay process, but also to the occurrence of nonradiative trapping with increasing temperature. They measured the temperature-dependent decay lifetimes of the CDs and shown in Fig. 12a. The data were collected by monitoring emission maximum as a function of the temperature under the 320 nm laser excitation. The result shows that the PL lifetime drops from 15.03 to 11.70 ns with the temperature increasing from 283 to 343 K, which could be ascribed to the occurrence of nonradiative decay processes. Besides, the PL relaxation dynamics of the CDs reveal multi-exponential decay with temperature increasing, which suggests the photoexcited carriers following the complicated relaxation processes. The occurrence of non-radiative trapping will be increased with rising temperature, and this could be quantitatively analyzed by the Arrhenius plot of the integrated PL intensities as:

$$ I={I}_0/\left[1+\mathrm{a}\ \exp\ \left(\hbox{-} {\mathrm{E}}_a/\mathrm{kT}\right)\right] $$

where *E*_a_ is the activation energy, *k* is the Boltzmann constant, and *a* is a constant. Figure 12a displays the plotting of the emission intensity with respect to 1/*T*, where the value of activation energy (*E*_a_) is calculated to be 0.329 ± 0.02 eV. In order to probe the reason for thermal quenching of CDs emission process, the radiative (*V*_r_) and nonradiative (*V*_nr_) recombination rates of CDs were determined from the lifetime (τ*) and quantum yield (QY) as:

$$ {\tau}^{\ast }=\frac{1}{V\mathrm{r}+V\mathrm{nr}};\mathrm{QY}=\frac{V\mathrm{r}}{V\mathrm{r}+V\mathrm{nr}} $$

The QY of CDs at various temperatures was calculated from their temperature-dependent absorption and integrated PL intensity with the QY determined at room temperature. They have noticed that the radiative rates have a slight decline when the temperature rises from 283 to 343 K; at the same time, the nonradiative recombination rates have gradually increased by about 2-fold. These results further indicate that the temperature-activated PL quenching in CDs is mainly due to the activation of nonradiative relaxation channels [102].

Other groups used microscopic and spectroscopic techniques to understand the mechanism of thermos-sensing of carbon dots.

**Fig. 13:**
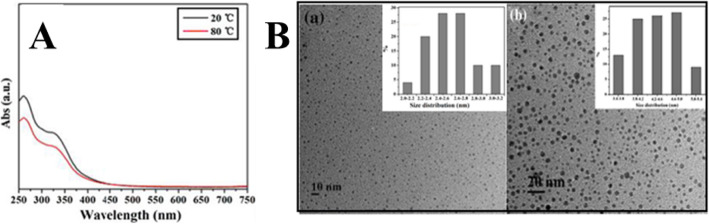
**a** UV–Vis absorption spectra of CDs in aqueous solution under 20 and 80 °C. **b** the TEM image of CDs in aqueous solution (**a**) at room temperature, the average size was 2.6 ± 0.2 nm (**b**) at 80 °C, and the size increased up to 4.4 ± 0.2 nm. (Reproduced from reference [1])

Wang at el. used TEM and UV–Vis spectra to study the temperature-responsive PL behavior of prepared CDs. As shown in Fig. 13a, the CDs display no change in the UV–Vis spectra upon increasing the temperature from 20 to 80 °C. However, it was found that the average diameter of CDs increased from 2.6 ± 0.2 nm at room temperature to 4.4 ± 0.2 nm at 80 °C (Fig. 13b). Thus, increasing the temperature, the aggregation of as-prepared CDs occurred which caused the obvious fluorescence quenching [1].

**Fig. 14:**
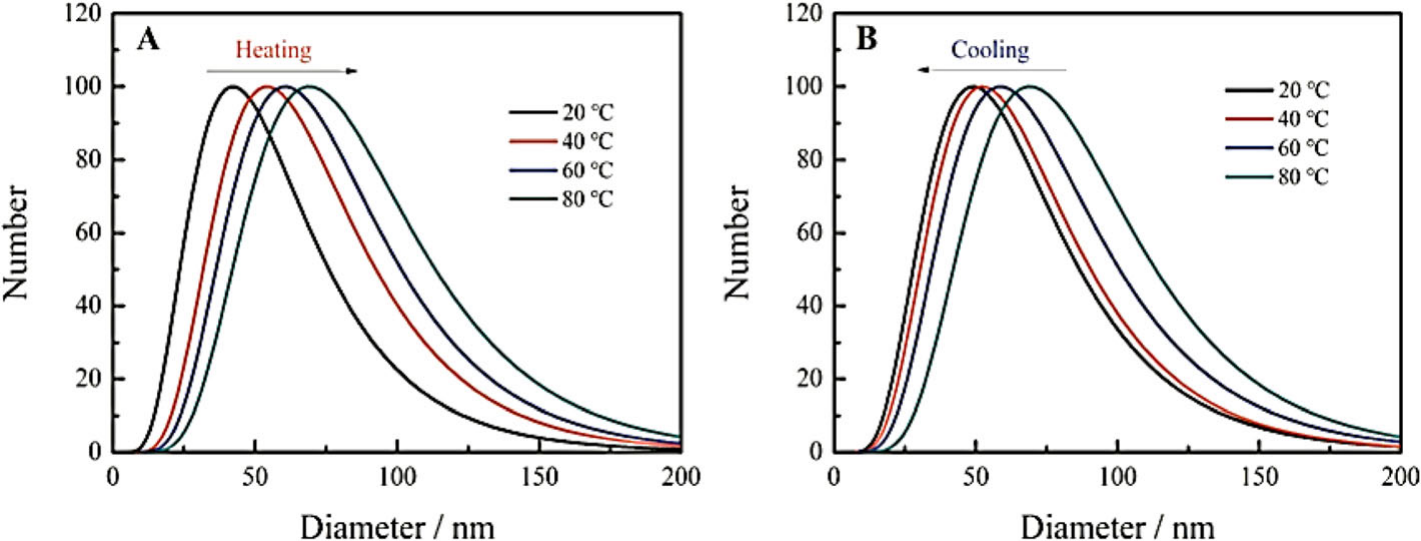
Change of hydrated particle size of carbon dots during heating (**a**) and cooling (**b**). (Reproduced from reference [101])

He et al. [101] reported that the hydration particle size of their CDs emerges as larger with the increase in the temperature (Fig. 14a), which indicates that the temperature rise gives rise to the aggregation of the CDs, eventually results in the fluorescence quenching. Nonetheless, with the decline in the temperature, the hydration particle size of CDs starts declining (Fig. 14b), which indicates that the cooling has the potential of causing CDs to depolymerize [101].

Another group such as Cui et al. also attributed the fluorescence quenching to the aggregation of CDs. They also tried to apply the undoped CDs synthesized using only acrylic acid as a precursor in temperature sensors. Unfortunately, undoped CDs possessed weaker quenching effects under the same temperature elevation than doped CDs [92].

**Fig. 15.**
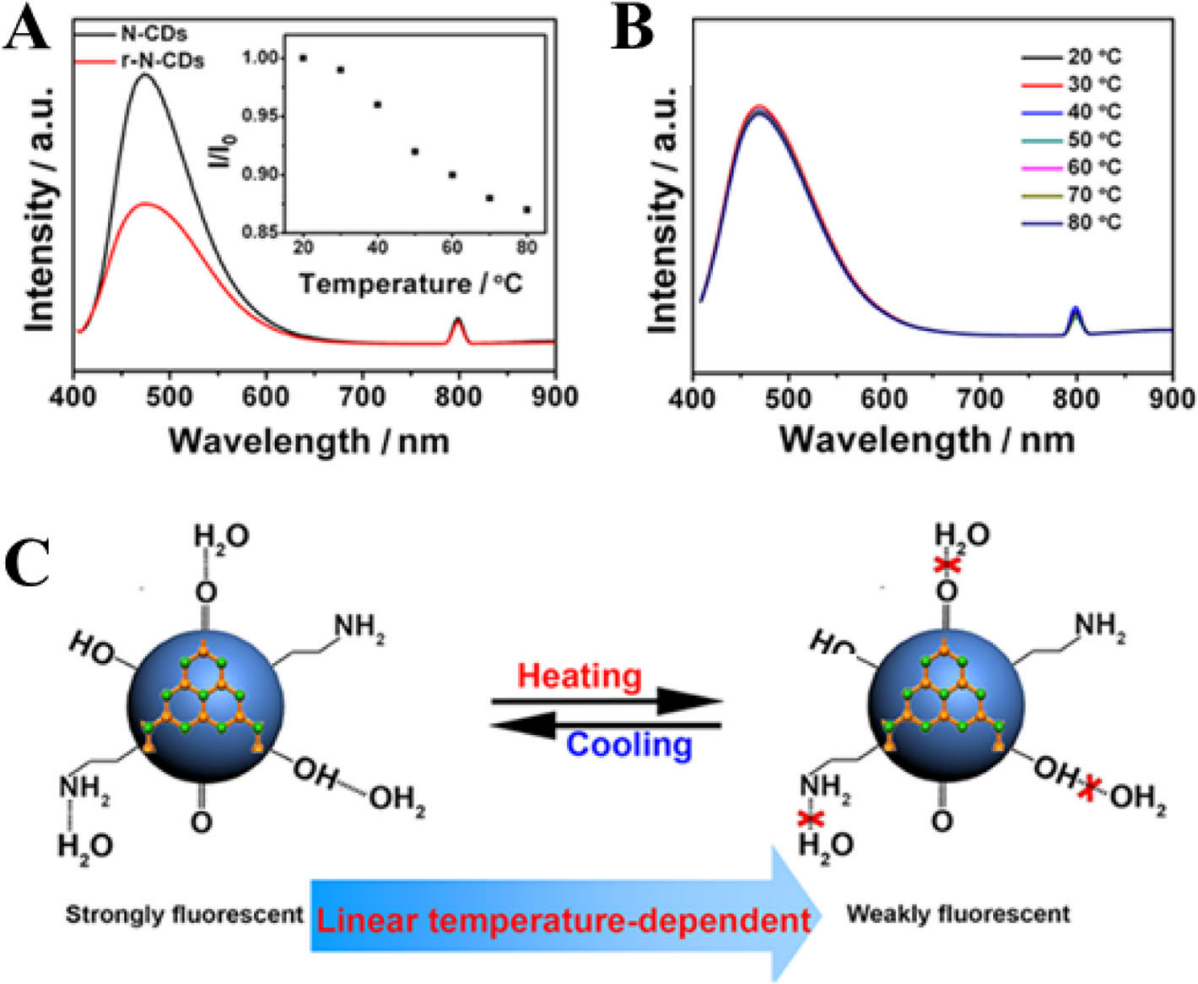
**a** PL spectra (excitation wavelength, 400 nm) of N-CDs (black trace) and r-N-CDs (red trace). The inset shows *I*/*I*_0_−T of reduced N-CDs. **b** PL spectra (excitation wavelength, 400 nm) of N-CDs dispersed in C_2_H_5_OH at various temperatures. **c** A schematic mechanism for the temperature-dependent fluorescence intensity of N-CDs. (Reproduced from reference [94])

Yang et al. [94] in their work proposed two key factors concerning the temperature-dependent PL property of the N-CDs, including (i) surface functional groups and (ii) hydrogen-bonding interaction. To examine the effect of the first factor, the surface O-containing groups, another control experiment was conducted by treating N-CDs (4.0 mL) with a strong reducing agent NaBH_4_ (1.0 mL, 0.1 mol L^−1^) to remove C=O species on carbon dots surface. The obtained reduced N-CDs are denoted as r-N-CDs for brevity. Compared with N-CDs, the r-N-CDs exhibit weaker fluorescence intensity (Fig. 15a). Besides, the fluorescence intensity of r-N-CDs only decreases by 13% with temperature increasing from 20 to 80 °C (the inset in Fig. 15a) that gives a much lower temperature sensitivity, which is ascribed to the decreased O-containing groups [94].

The second factor, the effect of hydrogen bonding with the solvent on fluorescent behavior of N-CDs was explored. N-CDs solution (1.0 mL) was dropped on a filter paper and left to dry in the air to obtain a solid sample, which still emits bright fluorescence. However, no obvious change of fluorescence intensity of the solid N-CDs with temperature increase was observed. They also measured the fluorescence of N-CDs dispersed in ethanol. The fluorescence intensity of N-CDs in C_2_H_5_OH is lower than that in water and little variation of the PL intensity is observed with temperature increasing from 20 to 80 °C (Fig. 15b). Hence, the strong hydrogen bonds play a key role in the temperature-dependent PL property of the N-CDs. Figure 15c is a schematic mechanism for the temperature-dependent fluorescence intensity of N-CDs [94].

**Fig. 16.**
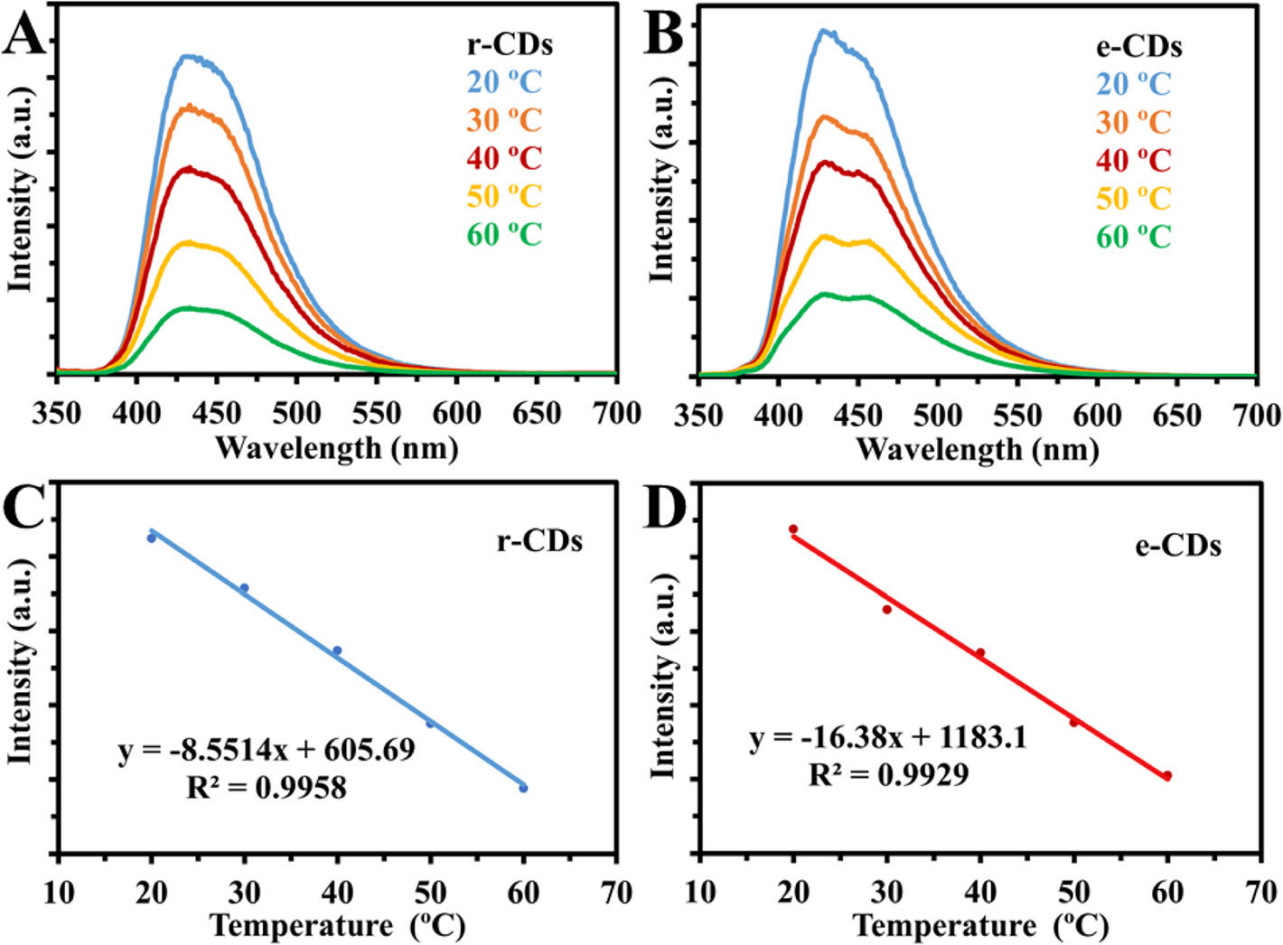
**a**, **b** Fluorescence spectra of reduced CDs (r-CDs) and CDs in ethanol (e-CDs) at temperatures (20 to 60 °C). **c**, d Linear correlation between fluorescence intensity and temperature (°C) for r-CDs and e-CDs respectively. (Reproduced from the supplementary information of reference [77])

However, our group used the same experimental strategy as Yang group; in both cases, the r-CDs and e-CDs emissions were quenched linearly with increasing temperature, in the same way as the original results of their CDs (Fig. 16). Thus, our results ruled out the synergistic effects of abundant oxygen-containing functional groups and hydrogen bonds [77].

## Bioimaging in Living Cells (Thermal Imaging)

In literature, only a few articles explored the temperature-responsive fluorescent properties of CDs in biological imaging. Prior to such experimentations, in vitro cytotoxicity analysis is crucial for CDs because they make it possible to estimate the CD’s toxicity in living subjects. In vitro cytotoxicity analysis evaluates the effect or influence of the nanomaterial on cultured cells [89].

**Fig. 17:**
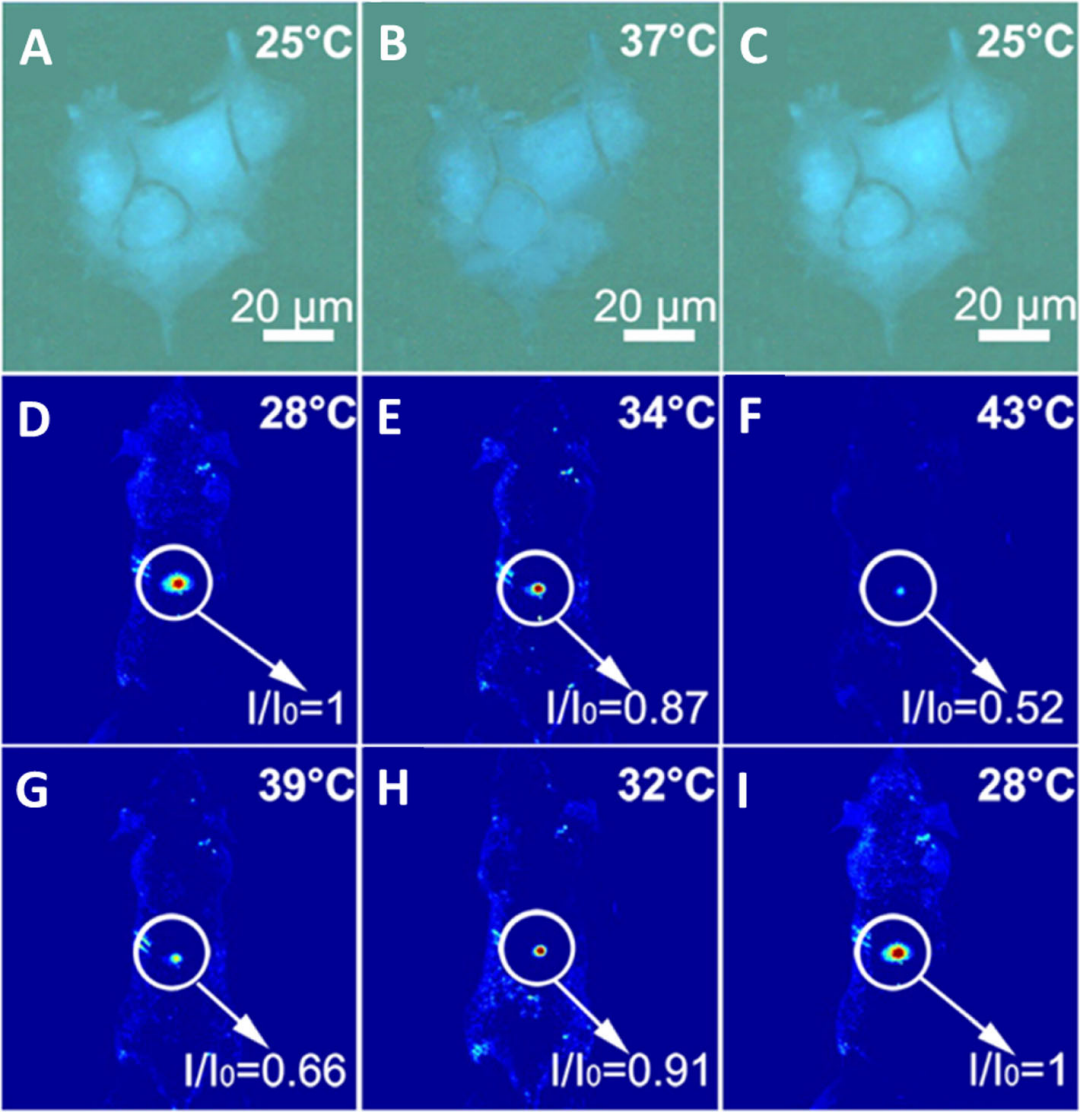
**a–c** Fluorescent images of a single Hela cell at 25, 37, and 25 °C after treatment with N-CDs, respectively. **d**–**f** Fluorescent photographs of a mouse given an injection of N-CDs at increasing temperatures. **g**–**i** Fluorescent photographs of a mouse given an injection of N-CDs at decreasing temperatures. ( Reproduced from reference [94])

Yang et al. [94] verified that the CDs could be used as an effective thermometer in living cells; HeLa cells were washed with PBS after treatment with CDs for 6 h. Bright-blue fluorescence of the CDs in HeLa cells is observed when the temperature is 25 °C as shown in Fig. 17a. With the temperature increasing to 37 °C, the blue fluorescence becomes weaker (Fig. 17b), the fluorescence of N-CDs is recovered when the temperature was decreased to 25 °C (Fig. 17c). As a thermos-imaging in vivo, the fluorescent images of mice were collected immediately after being injected with N-CDs at different temperatures. By setting the fluorescence intensity at 28 °C as the reference (*I*_o_), *I*/*I*_o_ of the area where CDs were injected varies from 1.0 to 0.87 with the increase of temperature from 28 to 34 °C (Fig. 17d, e). With temperature further increasing to 43 °C, *I*/*I*_o_ declines to 0.52 and the fluorescence becomes nearly undetectable (Fig. 17f). And *I*/*I*_o_ can be reversibly enhanced back from 0.66 to 1.0 with the temperature decreases from 39 to 28 °C (Fig. 17g–i). All of these results indicated that N-CD could be used as an effective in vitro and in vivo nanothermometer [94].

**Fig. 18:**
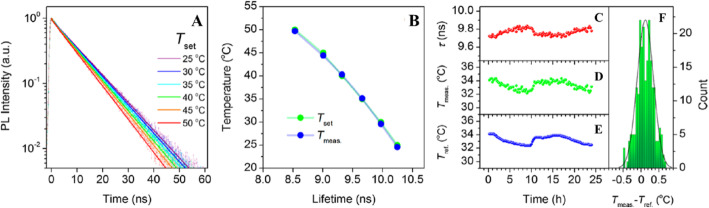
In vitro intracellular PL lifetime thermal sensing using CDs. **a** PL emission decays of HeLa cells incubated with CDs (500 μg/mL) at different temperatures (*T*_set_). **b** Temperatures determined using the calibration (*T*_meas_) and set temperatures (*T*_set_) plotted against the PL lifetime. **c**–**f** Applicability of CDs for long-term remote intracellular temperature monitoring. **c** PL lifetimes extracted from PL transients recorded every 15 min for 24 h of HeLa cells incubated with CDs (500 μg/mL). **d** Temperatures determined using the calibration curve. **e** Temperatures measured with a reference thermometer (*T*_ref_). **f** Histogram showing the distribution of temperature differences between the obtained and reference temperatures; the solid line is the distribution curve. (Reproduced from reference [89])

In an exploratory experiment, Kalytchuk et al. tested the capacity of CDs for intracellular temperature monitoring in human cervical cancer HeLa cells. Figure 18 shows the measured intracellular temperatures of HeLa cells incubated with CDs (500 μg/mL). The CDs’ PL decay curves at each temperature were highly reproducible and could be fitted with a single-exponential function at all recorded temperatures. In Fig. 18a, the recorded PL decay curves for temperatures between 25 and 50 °C (with a step size of 5 °C) are indicated by symbols, while the corresponding single-exponential fits are represented by solid lines. They were able to confirm that the PL signal in these PL lifetime measurements was derived exclusively from the CDs for the CD concentration of ≥ 100 μg/mL. The intracellular temperature in each measurement was determined from the calibration curve between PL lifetimes as the temperature increases from 2 to 80 °C. The temperatures determined in this way (*T*_meas_) are plotted as functions of the PL lifetime in Fig. 18b. Independently, the temperature of the cell solution was determined using a calibrated reference thermometer (shown in Fig. 18b as T_set_). The temperatures reported by the luminescent CD probe and the reference detector are in good agreement. These results show that the PL lifetime of nanoprobes based on CDs can be reliably used to measure intracellular temperatures [89].

To further evaluate the potential of luminescent CD nanoprobes for long-term real-time temperature monitoring, PL decay profiles of HeLa cells incubated with CDs (500 μg/mL) every 15 min for 24 h were recorded. The PL lifetimes extracted from the measured PL decay values during this period were then plotted as functions of time, as shown in Fig. 18c. Using these results, the temperature variation over time was calculated using the calibration curve, as shown in Fig. 18d. In addition, the sample’s temperature at each measurement point was determined using a reference thermometer with a temperature reproducibility of 60 mK. The temperature determined with the reference thermometer is plotted in Fig. 18e, which shows that there was excellent agreement between the measured and reference temperatures. The high accuracy of the PL-based temperature measurements is further demonstrated by statistical analysis of the differences between the measured and real (reference) temperatures (Fig. 18f). Using these data, the absolute average accuracy of temperature detection by the presented method was calculated to be 0.27 °C. This experiment confirms the potential of CD-based thermal probes in biological systems [89].

**Fig. 19:**
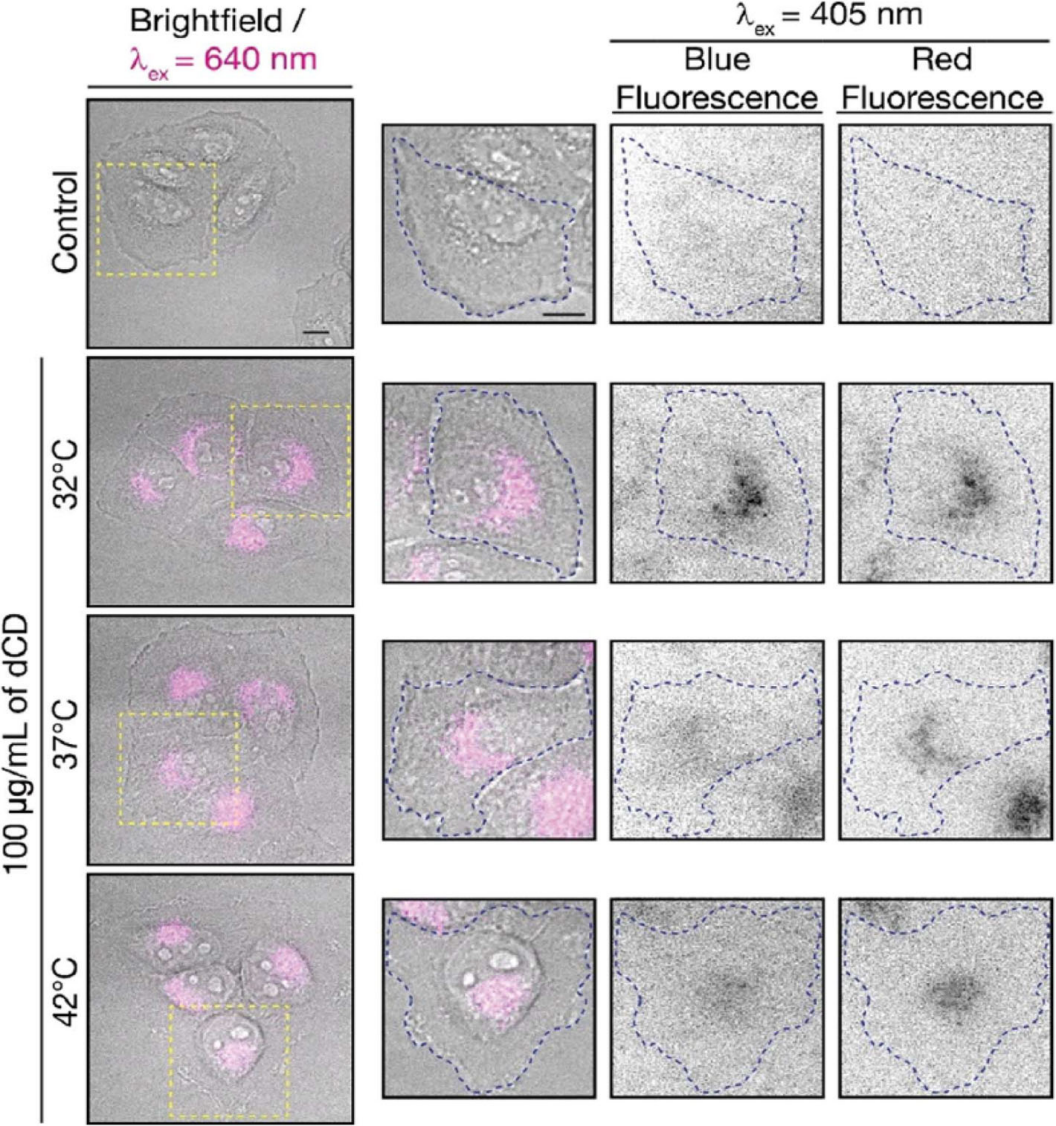
Fluorescence microscopy images of CD-treated HeLa cells. Fluorescence signals from the CDs (λ_ex_ = 640 nm; left and 405 nm; right) fluorescence ratios are 1.8 at 32 °C, 2.0 at 37 °C, and 2.3 at 42 °C. The control shows untreated HeLa cells at 42 °C with no fluorescence signal as expected. (Reproduced from reference [29])

Macairan et al. [29] displayed that the prepared CDs can be used for thermal sensing inside cells using intensity and ratiometric approaches. HeLa cells treated with CDs were allowed to equilibrate at 32, 37, and 42 °C (Fig. 19). Excitation at 640 nm was used to selectively monitor the red fluorescence of the CDs in the cells. The thermal changes could be due to changes in intracellular concentration or not correlate with a change in intensity (λex + 640 nm). This could be due to changes in intracellular concentration or localization of the CDs at higher temperatures. Thus, simply relying on changes in fluorescence intensity leads to accurate intracellular thermal sensing.

In contrast, these limitations are excluded using the ratiometric approach. The CDs maintain dual blue and red fluorescence in cells following excitation at 405 nm, as previously observed for the colloidal dispersions (Fig. 19). The red-to-blue ratio increases with increasing temperature, with values of 1.8 at 32 °C, 2.0 at 37 °C, and 2.3 at 42 °C. The ratiometric relationship of the red-to-blue fluorescence of the CDs highlights the advantage of ratiometric temperature sensing in the development of fluorescent nano-thermometry probes. The relative red-to-blue emission ratio remains unaffected, regardless of the amount of CDs taken up by the cells, which can be affected by various factors such as confluency and is not concentration-dependent. Lastly, the CDs have shown fluorescence reversibility with respect to changes in intracellular temperature. Following incubation in HeLa cells, they were subjected to a heating/cooling cycle from 32/42/32 °C. This emphasizes the robustness of the proposed CD-nanothermometer and these findings further demonstrate the fluorescence reversibility [29].

**Fig. 20:**
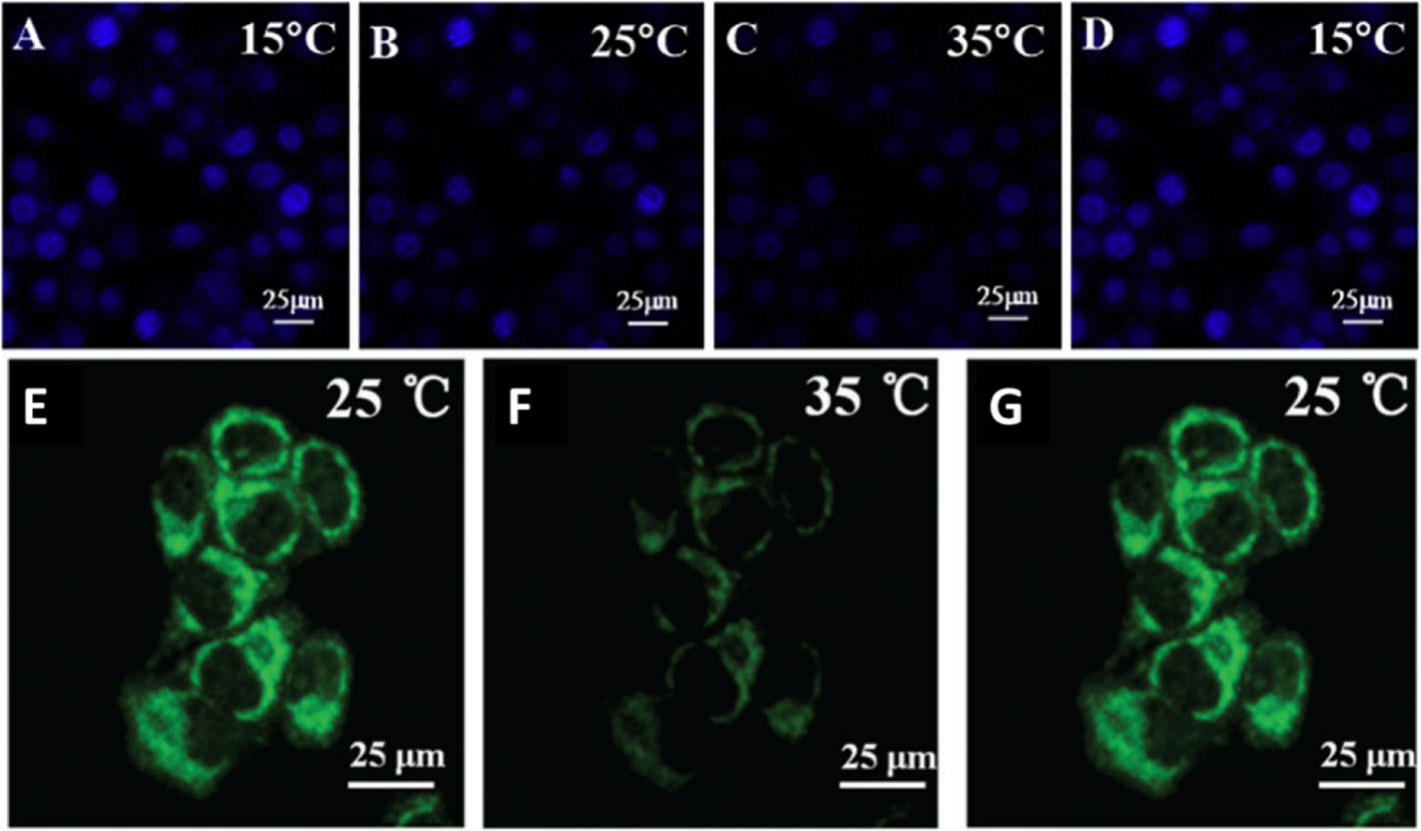
**a–d** Are confocal microscopy images of N, S, I-CDs-colon cancer cell HT-29 with corresponding fluorescence field at 15, 25, 35, and 15 °C, respectively. (Reproduced from reference [100]). **e**–**g** Confocal microscopy images of N, S-CDs-stained cells with corresponding fluorescence field at 25, 35, and 25 1C, respectively. (Reproduced from reference [96])

Confocal laser scanning microscopy was used to thermal image colon cancer cell HT-29 using N, S, and I-doped CDs, as shown in Fig. 20 [100]. The fluorescent spots were temperature-dependent as shown in Fig. 20g–i, as the most intense is at 15 °C, while the weakest point was at 35 °C. Interestingly, the fluorescence spots were reversible, and the spots were very photostable after 20 min of continuous excitation [100]. Shin et al. [96] also used confocal laser scanning microscopy for Hela cells, shown in Fig. 20e–g.

**Fig. 21:**
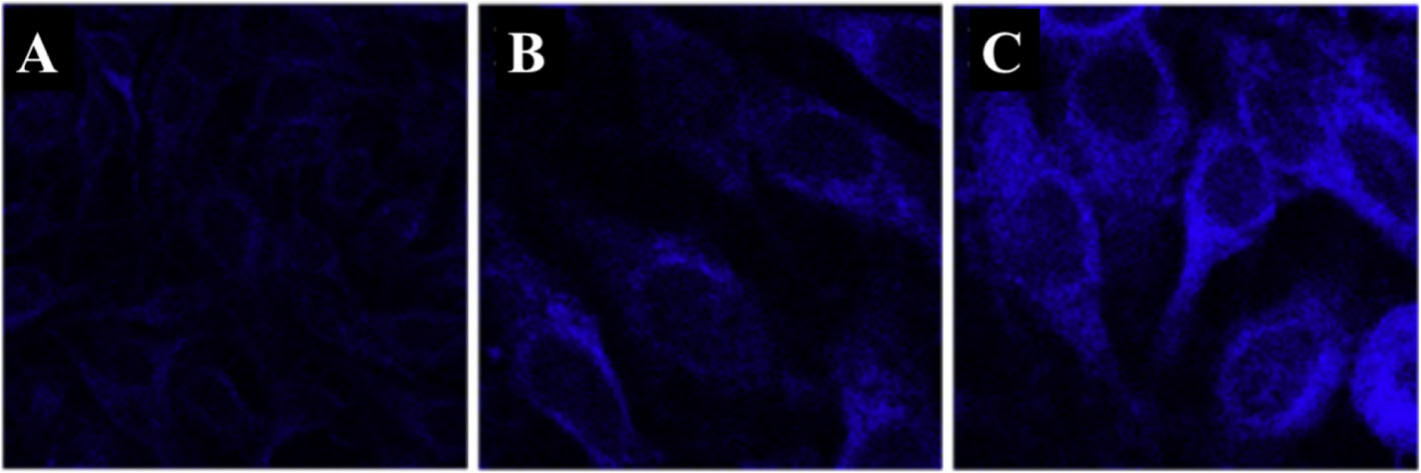
Confocal laser scanning microscopy images (excited at 405 nm) of MnOx-CDs in HepG2 cells at 40 °C (**a**), at 30 °C (**b**), and 20 °C (**c**). (Reproduced from reference [103])

Li et al. 103 prepared a nanocomposite composed of MnOx-CDs to be used as a nano thermos responsive fluorophore for biological environments. HepG2 cells were incubated with MnOx-CDs at different culture temperatures. As illustrated by confocal laser scanning microscopy (excited at 405 nm), the blue luminescence of the MnOx-CDs in the HepG2 cells is weak when the environmental temperature was 40 °C (Fig. 21a), and the blue luminescence of the MnOx-CDs in HepG2 cells enhances as the temperature decreased to 30 °C (Fig. 21b) even to 20 °C (Fig. 21c). Due to the temperature-responsive properties, the as-synthesized MnOx-CDs can be readily applied in the biomedical fields like bioimaging and photothermal therapy in cancer treatment [103].

## Conclusions and Future Perspective

Carbon nanodots exhibit unique properties to be exploited for nanothermometry, such as thermal-sensitivity, low-cost, and photostability. Flexible surface modification and facile preparation will pave the way to establish an enormous number of thermal sensitive nanomaterials for a variety of applications. The overall trends in thermo-sensing nanomaterials are aimed at enhancing photostability and thermal-resolution with using low-cost and safe materials. CDs can be classified as a new generation of thermometer that can fulfill these requirements and can be used for biomedical thermometry applications, such as temperature monitoring during hyperthermia treatment. Facile-preparation protocols, biocompatibility, and easy functionalization of CDs are promising criteria which make the CDs alternative next-generation nanothermometer materials. More efforts are required to promote basic research in this field. Limitations should be overcome to produce carbon dot-based nanothermometers comprising enhancing thermal sensitivity, and working in a broader range of temperature. A better understanding of the fluorescence thermal-sensing mechanism is another key issue to be able to design and manipulate the structure of CDs and enhance thermal resolution. More experiments and theoretical modeling are necessary to understand the correlation between the methods of fabrication of CDs with their thermal behavior.
